# Engineered *Corynebacterium glutamicum* as the Platform for the Production of Aromatic Aldehydes

**DOI:** 10.3389/fbioe.2022.880277

**Published:** 2022-05-12

**Authors:** Hyun-Song Kim, Jung-A Choi, Bu-Yeon Kim, Lenny Ferrer, Jung-Min Choi, Volker F. Wendisch, Jin-Ho Lee

**Affiliations:** ^1^ Department of Food Science and Biotechnology, Kyungsung University, Busan, South Korea; ^2^ Genetics of Prokaryotes, Faculty of Biology and Center for Biotechnology, Bielefeld University, Bielefeld, Germany

**Keywords:** aromatic aldehyde reductase, NCgl0324, aromatic aldehydes, microbial production, *Corynebacterium glutamicum*

## Abstract

Aromatic aldehydes, including 4-hydroxybenzaldehyde (4-HB aldehyde), protocatechuic (PC) aldehyde, and vanillin, are used as important flavors, fragrances, and pharmaceutical precursors and have several biological and therapeutic effects. Production of aromatic aldehydes in microbial hosts poses a challenge due to its rapid and endogenous reduction to alcohols. To address this hurdle, prospecting of the genome of *Corynebacterium glutamicum* yielded 27 candidate proteins that were used in comprehensive screening with a 4-hydroxybenzyl (4-HB) alcohol–producing strain. We identified that NCgl0324 has aromatic aldehyde reductase activity and contributed to 4-HB aldehyde reduction *in vivo* since the *NCgl0324* deletion strain HB-*Δ0324* produced 1.36 g/L of 4-HB aldehyde, that is, about 188% more than its parental strain. To demonstrate that *NCgl0324* knockout can also improve production of PC aldehyde and vanillin, first, a basal MA303 strain that produces protocatechuate was engineered from 4-hydroxybenzoate-synthesizing *C. glutamicum* APS963, followed by deletion of *NCgl0324* to generate PV-*Δ0324*. The PC aldehyde/alcohol or vanillin/vanillyl alcohol biosynthetic pathways, respectively, were able to be expanded from protocatechuate upon introduction of carboxylic acid reductase (CAR) and catechol *O*-methyltransferase encoded by a mutated *comt* ^m^ gene. In shake flask culture, the resulting *NCgl0324* deletion strains PV-I*Δ0324* and PV-IY*Δ0324* were shown to produce 1.18 g/L PC aldehyde and 0.31 g/L vanillin, respectively. Thus, modulation of the identified *NCgl0324* gene was shown to have the potential to boost production of valuable aromatic aldehydes and alcohols.

## Graphical Abstract

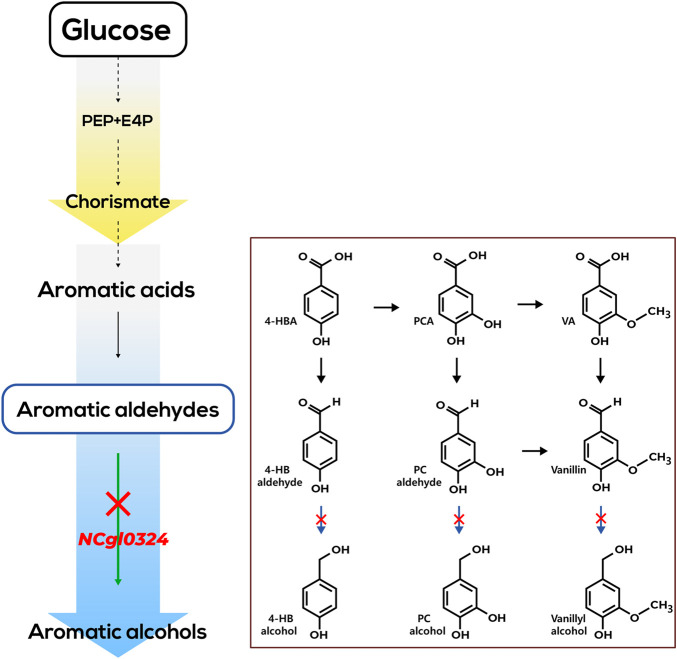

## Introduction

Aromatic aldehydes, such as benzaldehyde, 4-hydroxybenzaldehyde (4-HB aldehyde), protocatechuic aldehyde (PC aldehyde), vanillin, cuminaldehyde, cinnamic aldehyde, and syringic aldehyde, are used as flavors, fragrances, and pharmaceutical precursors (Kunjapur et al., 2014; Kunjapur and Prather, 2015). In addition, they are described to have many useful biological activities and therapeutic effects. For example, aside from its use as a flavoring and fragrance agent (Krings and Berger, 1998), benzaldehyde also possesses antimicrobial activities against food poisoning bacteria (Bowles and Juneja, 1998). 4-HB aldehyde has been proposed to be a candidate therapeutic agent with the potential to promote acute wound healing (Ha et al., 2000; Kang et al., 2017). PC aldehyde possesses antiproliferative, antioxidant, antiadipogenic, and anticancer properties (Byun et al., 2016; Zhong et al., 2016; Shi Zhong et al., 2020; Zhiwei Zhong et al., 2020). Vanillin is the primary component of the extract of vanilla bean and considered one of the most important aromatic aldehydes widely used in food, beverage, pharmaceutical, and other applications (Martău et al., 2021). Most of vanillin on the market is produced by chemical synthesis from guaiacol and glyoxylic acid (Martău et al., 2021). Many aromatic aldehydes can be naturally extracted from plants or fungi, for example, 4-HB aldehyde from *Gastrodia elata* (Ha et al., 2000; Zhan et al., 2016), PC aldehyde from *Salvia miltiorrhiza* (Byun et al., 2016) and *Phellinus gilvus* (Zhong et al., 2016), and vanillin from *Vanilla vanilifola* (Gallage and Møller, 2015). Due to excessive exploitation of plant resources, little content of aromatic aldehydes, seasonal variations, complicated extraction and purification processes, and microbial production of aromatic aldehydes are considered a promising alternative (Krings and Berger, 1998; Bai et al., 2016; Lee and Wendisch, 2017; Cravens et al., 2019; Martău et al., 2021).

With the advent of synthetic biology, systems metabolic engineering allowed to endow microbes with artificial metabolic pathways for the production of value-added aromatic aldehydes that cannot be produced by wild-type microbes (Hansen et al., 2009; Kunjapur and Prather, 2015). As an illustrative example, *de novo* biosynthesis of vanillin from d-glucose *via* 3-dehydroshikimate was achieved by the introduction of 3-dehydroshikimate dehydratase, carboxylic acid reductase (CAR), phosphopantetheinyl transferase, and catechol *O*-methyltransferase (COMT) in the yeasts *Saccharomyces cerevisiae* and *Shizosaccharomyces pombe* (Hansen et al., 2009). However, accumulation of vanillin in yeasts was limited by many endogenous-related enzymes. This hurdle was overcome by inactivation of the *ADH6*-encoded alcohol dehydrogenase (ADH) among tested 29 known or hypothetical ADHs, aryl-ADHs, and related aldehyde reductases, resulting in a 50% decrease in converting vanillin to vanillyl alcohol. Minimizing endogenous conversion of aldehydes to corresponding alcohols has also been studied in *Escherichia coli* for aldehyde production. Kunjapur et al. (2014) demonstrated that the deletion of six genes, *dkgA*, *dkgB*, *yeaE*, *yahK*, *yjgB* (*ahr*), and *yqhD*, in *E. coli* (RARE strain) can lead to production of commercially interesting aromatic aldehydes, including benzaldehyde and vanillin, with minimal formation of alcohols. Rodriguez and Atsumi (2014) reported that combined deletion of 13 genes, that is, *adhE*, *yqhD*, *adhP*, *eutG*, *yiaY*, *ahr*, *betA*, *fucO*, *yahK*, *dkgA*, *gldA*, *ybbO*, and *yghA* in *E. coli* (AL1728 strain) resulted in a 90–99% reduction in endogenous aldehyde reduction activity for a wide range of aliphatic aldehyde substrates ranging from C2 to C12. Given that the pivotal genotype of the RARE strain overlaps with the genotype of the AL1728 strain, both strains probably produce useful aromatic and aliphatic aldehydes (Kunjapur and Prather, 2015). In a related study about transcriptional analysis of *Corynebacterium glutamicum* on toxic furfuraldehyde and benzaldehyde exposure, 51 genes encoding alcohol dehydrogenases, aldehyde dehydrogenases, and other oxidoreductases represented significantly upregulated RNA levels (Zhou et al., 2019). Of these, three ADH genes including *CGS9114_RS01115*, *CGS9114_RS10340*, and *CGS9114_RS09230*; two putative oxidoreductase genes including *CGS9114_RS11565* and *CGS9114_RS06005*; and one multicopper oxidase gene *CGS9114_RS09375* seem to be closely associated with the biotransformation of several aldehydes in *C. glutamicum*. On the one hand, Tsuge et al. (2016) showed that FudC encoded by *NCgl0324* from *C. glutamicum* functions as a dehydrogenase with higher specificity toward furfural than for acetaldehyde and isobutyraldehyde and is involved in furfural detoxification.

**FIGURE 1:**
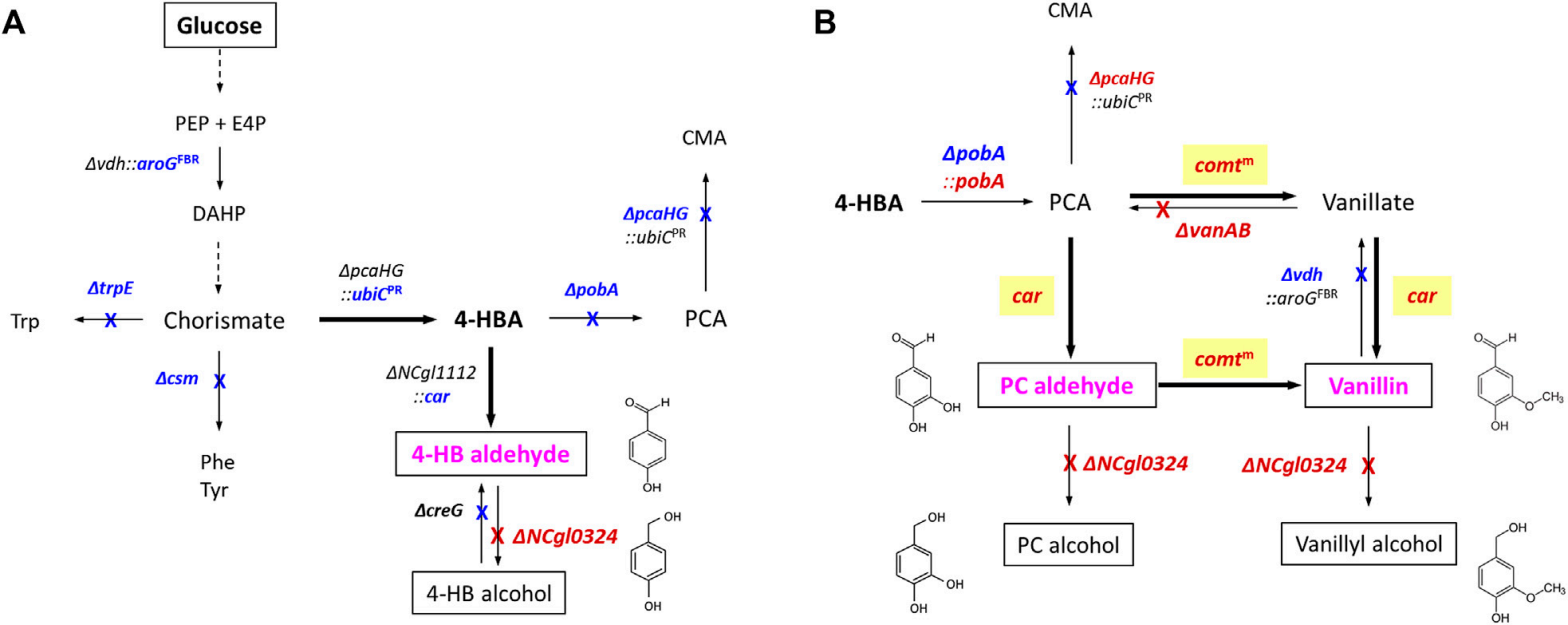
Schematic representation of artificial *de novo* biosynthetic pathways of 4-hydroxybenzaldehyde. **(A)**, protocatechuic aldehyde, and vanillin **(B)** in *C*. *glutamicum*. Bold arrows represent artificial catalytic steps by heterologous expression of corresponding genes; crosses indicate the disruption of corresponding genes. The abbreviations are as follows: PEP, phosphoenolpyruvate; E4P, erythrose 4-phosphate; DAHP, 3-deoxy-d-arabinoheptulosonate-7-phosphate; Phe, l-phenylalanine; Tyr, l-tyrosine; Trp, l-tryptophan; 4-HBA, 4-hydroxybenzoate; 4-HB aldehyde, 4-hydroxybenzaldehyde; 4-HB alcohol, 4-hydroxybenzyl alcohol; PCA, protocatechuate (3,4-dihydroxybenzoate); CMA, β-carboxymuconate; PC aldehyde, protocatechuic aldehyde; PC alcohol, protocatechuic alcohol. Genes and corresponding enzymes are as follows: *vdh*, vanillin dehydrogenase; *aroG* ^FBR^, a feedback-resistant DAHP synthase from *E. coli*; *trpE*, anthranilate synthase; *csm*, chorismate mutase; *pcaHG*, protocatechuate dioxygenase; *ubiC* ^PR^, a product-resistant chorismate-pyruvate lyase from *E. coli*; *pobA*, 4-hydroxybenzoate hydroxylase; *car*, carboxylic acid reductase from *Nocardia iowensis*; *creG*, NAD^+^-dependent dehydrogenase; *comt* ^m^, a mutant catechol *O*-methyltransferase (Y200L COMT) from *Rattus norvegicus*; *vanAB*, vanillate demethylase subunits **A** and **B**

*C. glutamicum* is used as a host organism for GRAS (generally regarded as safe) food and feed products and is the workhouse of fermentative production of amino acids, for example, l-glutamate and l-lysine (Wendisch, 2020). A plethora of metabolic pathways for breakdown and assimilation of aromatic compounds are known in *C. glutamicum*, which guided the biosynthesis of versatile aromatic compounds by interception and/or extension of these pathways (Brinkrolf et al., 2006; Shen et al., 2012; Lee and Wendisch, 2017). Thus, this bacterium has been used a prominent host for the production of aromatic compounds, such as l-tryptophan, halogenated l-tryptophans, anthranilate, methylated anthranilates, 4-hydroxybenzoate (4-HBA), 4-aminobenzoate, protocatechuate (PCA), indole-3-acetic acid, violacein, and anthocyanin (Lee and Wendisch, 2017; Kim et al., 2019; Wendisch et al., 2022). Recently, we engineered *C. glutamicum* to produce 19 g/L of 4-HBA in a 5-L bioreactor (Syukur Purwanto et al., 2018), and, by extension of this concept, introduction of CAR enabled production of 2.3 g/L of 4-HB alcohol as a main product and 0.3 g/L of 4-HB aldehyde as a minor by-product in flask culture (Kim et al., 2020; Figure 1A). The finding that more 4-HB alcohol than 4-HB aldehyde accumulated might be explained by the yet undescribed endogenous aldehyde reductase(s). Hence, systematic deletion of genes encoding these enzymes appears as a reasonable strategy to improve accumulation of 4-HB aldehyde. Moreover, due to their structural similarities, we expected that production of PC aldehyde or vanillin may be realized by extending metabolic pathways from the 4-HB aldehyde–producing strain **(** Figure 1B **)**. However, despite their importance for aromatic aldehyde production, little is known about genes and enzymes involved in reduction of aromatic aldehydes in *C. glutamicum*. With the aim of constructing strains producing 4-HB aldehyde, PC aldehyde, or vanillin based on a 4-HBA–producing *C. glutamicum*, gene mining for 4-HB aldehyde reduction is a pivotal strategy. In a systematic screening approach, we explored and identified genes responsible for 4-HB aldehyde reduction by the 4-HB alcohol–producing strain *C. glutamicum* GAS355. Upon deletion of *NCgl0324*, higher titers of 4-HB aldehyde, PC aldehyde, or vanillin were produced, while titers for the corresponding alcohols 4-HB alcohol, PC alcohol, or vanillyl alcohol were decreased **(** Figure 1 **)**. The results prove that modulation of the identified gene, *NCgl0324*, can boost development of the platform strain for the production of value-added versatile aromatic aldehydes and alcohols.

## Materials and Methods

### Bacterial Strains, Plasmids, and Molecular Genetic Techniques

Bacterial strains and plasmids used in this study are listed in Table 1 and Supplementary Table S1. *E. coli* TOP10 was used for plasmid construction. The *E. coli*/*C. glutamicum* shuttle vectors pCES208 and pCXE50 were used for gene expression in *C. glutamicum* (Lee, 2014; Syukur Purwanto et al., 2018). The plasmid pK19*mobsacB* was used for constructing deletion and insertion mutants of *C. glutamicum* (Schäfer et al., 1994). DNA fragments were amplified by polymerase chain reaction (PCR) using TOPsimple™ DryMIX-Tenuto (Enzynomics, Daejeon, Korea). An EZ-fusion^TM^ HT Cloning Kit (Enzynomics) was used for cloning of multiple DNA fragments *via* Gibson assembly (Gibson et al., 2009). The sequence correctness of PCR products cloned in vectors was confirmed by sequencing. Transformation of *C. glutamicum* was performed by electroporation (Eggeling and Bott, 2005).

**TABLE 1:** Bacterial strains and plasmid list used in this study.

Strain or plasmid	Characteristics	Source
*Escherichia coli*		
TOP10	*F* ^*−*^ *mcrA ∆(mrr-hsdRMS-mcrBC) φ80lacZ∆M15 ∆lacX74 recA1 endA1 araD139 ∆(ara-leu)7679 galU galK rps(StrR) endA1 nupG*	Invitrogen
BL21(DE3)	*F* ^*−*^ *dcm ompT hsdS(rB-mB-) gal λ(DE3)*	Novagen
*Corynebacterium glutamicum*		
ATCC 13032	Wild-type	ATCC
APS963	*ΔtrpE Δcsm ΔpobA Δvdh::*P_*ilvC-*_ *aroG* ^FBR^ P_*tuf*_ *aroC ΔqsuABCD::*P_*tuf*_ *-qsuC ΔbenABC::*P_*tuf*_ *-aroE*-T_*rrnB*_ *ΔNCgl2922::*P_*tuf*_ *-aroK-*T_*rrnB*_	Kim et al. (2020)
GAS355	APS963 *ΔNCgl1112::*P_*ilvC-M1*_ *-car-*T_*rrnB*_ *ΔcreG ΔpcaHG::*P_*sod*_ *-ubiC* ^pr^-T_*T7*_	Kim et al. (2020)
HB-*Δ0324*	GAS355 *ΔNCgl0324*	This study
MA183	APS963 *ΔpobA::pobA*	This study
MA225	MA183 *ΔpcaHG::*P_*sod*_ *-ubiC* ^pr^-T_*T7*_	This study
MA303	MA225 *ΔvanAB*	This study
MA-I303	MA303-harboring pICA4335	This study
MA-IY303	MA303-harboring pICA4335 and pYLS2250	This study
PV-*Δ0324*	MA303 *ΔNCgl0324*	This study
PV-I*Δ0324*	PV-*Δ0324*-harboring pICA4335	This study
PV-IY*Δ0324*	PV-*Δ0324*-harboring pICA4335 and pYLS2250	This study
Plasmid		
pK19*mobsacB*	Vector for allelic exchange in *C. glutamicum* (pK19*oriV*_*E.coli*_ *sacBlacZα*); 5.72 kb, Kan^R^	Schäfer et al. (1994)
pK19-*Δ0324*	pK19*mobsacB* derivative, 7.44 kb; 1.76 kb *NCgl0324* up and downstream fragments	This study
pK19-*pobA*	pK19*mobsacB* derivative, 8.06 kb; 1.19 kb *pobA* and 1.2 kb up and downstream fragments	This study
pK19-Δ*pcaHG*::P_*sod*_-*ubiC* ^pr^	pK19*mobsacB* derivative, 7.5 kb; 0.4 kb of the upstream region of *pcaH*, 1 kb P_*sod*_-*ubiC* ^pr^-T_*T7*_, and 0.4 kb of the downstream region from 97th nucleotide of *pcaG* ORF	Kim et al. (2020)
pK19-*ΔvanAB*	pK19*mobsacB* derivative, 6.76 kb; 1.1 kb *vanAB* up and downstream fragments	This study
pCXE50	*E. coli*/*C. glutamicum* expression shuttle vector; pCXM48 derivative with P_*tuf*_, T_*rrnB*_; 5.51 kb, pGA1 *ori*V_Cg_, Cm^R^	Syukur Purwanto et al. (2018)
pYL200	pCXE50 derivative with a mutant *comt* ORF (0.6 kb, Y200L) from *Rattus norvegicus*	This study
pYL230	pCXE50 derivative with a insertional mutation in TIR and a mutated *comt* ORF (0.6 kb) from *Rattus norvegicus*	This study
pYL250	pCXE50 derivative with a mutated *comt* ORF and a truncated *rrnB* transcriptional terminator (T_*rrnB*s_ 0.25 kb)	This study
pCES208	*E. coli*/*C. glutamicum* expression shuttle vector; 5.93 kb, Kan^R^	Syukur Purwanto et al. (2018)
pYLS2250	pCES208 derivative with 1.2 kb of P_*tuf*_ *-comt-*T_*rrn*_	This study
pICA4335	pCXI43 with *car* from *Nocardia iowensis*; 9 kb	Kim et al. (2020)
pXT-0324	pCXE50 derivative; expression of *NCgl0324* from *C. glutamicum*	This study
pET-0324	pET24a(+) derivative; expression of *NCgl0324* from *C. glutamicum*	This study

### Plasmid Construction for Chromosomal Gene Deletion and Insertion

The primers for deletion and insertion of genes are listed in Supplementary Tables S2, S3. To construct gene knockout plasmids responsible for known or hypothesized ADHs, oxidoreductases, aldo/keto reductases, *etc*., the primer sets for amplifying two flanking regions of 20 genes are as follows: P1/P2 and P3/P4 for *NCgl0186*, P7/P8 and P9/P10 for *NCgl0219*, P13/P14 and P15/P16 for *NCgl0313*, P19/P20 and P21/P22 for *NCgl0324*, P25/P26 and P27/P28 for *NCgl0503*, P31/P32 and P33/P34 for *NCgl0908*, P37/P38 and P39/P40 for *NCgl1213*, P43/P44 and P45/P46 for *NCgl1302*, P49/P50 and P51/P52 for *NCgl1459*, P55/P56 and P57/P58 for *NCgl1608*, P61/P62 and P63/P64 for *NCgl 1962*, P67/P68 and P69/P70 for *NCgl2122*, P73/P74 and P75/P76 for *NCgl2213*, P79/P80 and P81/P82 for *NCgl2277*, P85/P86 and P87/P88 for *NCgl2358*, P91/P92 and P93/P94 for *NCgl2382*, P97/P98 and P99/P100 for *NCgl2449*, P103/P104 and P105/P106 for *NCgl2582*, P109/P110 and P111/P112 for *NCgl2709*, and P115/P116 and P117/P118 for *NCgl2952*. The two amplified fragments of each gene were cloned into pK19*mobsacB*/*Hin*dIII/*Eco*RI using Gibson assembly, resulting in the construction of pK19-*Δ0186*, pK19-*Δ0219*, pK19-*Δ0313*, pK19-*Δ0324*, pK19-*Δ0503*, pK19-*Δ0908*, pK19-*Δ1213*, pK19-*Δ1302*, pK19-*Δ1459*, pK19-*Δ1608*, pK19-*Δ1962*, pK19-*Δ2122*, pK19-*Δ2213*, pK19-*Δ2277*, pK19-*Δ2358*, pK19-*Δ2382*, pK19-*Δ2449*, pK19-*Δ2582*, pK19-*Δ2709*, and pK19-*Δ2952* **(** Table 1 **)**.

To restore *pobA* from APS963, *pobA* gene (1.2 kb) and upstream and downstream regions (1.2 kb) were obtained from genomic DNA of *C. glutamicum* ATCC 13032 *via* PCR using the primer sets P-pobA-F and P-pobA-R. The amplified fragment was cloned to *Hin*dIII/*Xba*I-treated pK19*mobsacB* using Gibson assembly to generate pK19-*pobA*. The plasmid pK19-*ΔvanAB* was constructed by fusion of PCR fragments obtained by using primer sets P-vanA-F/P-vanA-R and P-vanB-F/P-vanB-R with *Hin*dIII/*Eco*RI-digested pK19*mobsacB* **(** Table 1 **)**.

### Plasmid Construction for Gene Expression

To construct a plasmid for the overexpression of the native *NCgl0324* gene in *C. glutamicum*, the *NCgl0324* ORF (open reading frame) from *C. glutamicum* was amplified *via* PCR using primer sets P-0324-F and P-0324-R and *C. glutamicum* genomic DNA as a template and then assembled with *Eco*RI- and *Hin*dIII-digested pCXE50, yielding pXT-0324 **(** Table 1 **)**. The plasmid for purification of NCgl0324 protein in *E. coli* was constructed as follows. The *NCgl0324* gene was achieved *via* PCR using the primer sets P-0324-NF and P-0324-XR and cloned in pET-24a(+) cut with *Nde*I and *Xho*I. The resulting plasmid was designated pET-0324 **(** Table 1 **)**. The plasmid construction for expressing a mutated *comt* gene from *Rattus norvegicus* encoding the soluble catechol *O*-methyltransferase mutant (Y200L COMT; accession number NP_036,663) is as follows (Law et al., 2016): a codon-optimized *comt* (0.666 kb) from *Rattus norvegicus* was synthesized from Integrated DNA Technologies (IDT, Iowa, United States; Supplementary Table S4) and cloned in pCXE50/*Eco*RI/*Hin*dIII, resulting in pYL200 **(** Table 1 **)**. To overexpress this gene in *C. glutamicum*, a plasmid with insertional mutation in the TIR (translation initiation region) in front of *comt* ORF was constructed. The amplified *tuf* promoter by PCR using primers P-X48-F and P-Ptuf-mR was digested with *Xba*I and *Eco*RI and ligated with pYL200/*Xba*I/*Eco*RI to gain the pYL230 plasmid. In addition, the plasmid pYL250 with the truncated *rrnB* transcriptional terminator (T_t-*rrnB*_) was constructed by insertion of the 0.23-kb truncated T_t-*rrnB*_ (primers P-TrrnB-tF and P-X48-R) into the pYL200/*Hin*dIII/*Kpn*I site. In order to express *comt* and *car* genes in different compatible vectors, the 1.2-kb fragment with *tuf* promoter, *comt* ORF, and T_t-*rrnB*_ in pYL250 was subcloned into the pCES208/*Not*I/*Kpn*I site, yielding the pYLS2250 plasmid **(** Table 1 **)**.

### Strain Construction for Deletion of Putative Aldehyde Reductase Genes

The plasmids pK19-*Δ0186*, pK19-*Δ0219*, pK19-*Δ0313*, pK19-*Δ0324*, pK19-*Δ0503*, pK19-*Δ0908*, pK19-*Δ1213*, pK19-*Δ1302*, pK19-*Δ1459*, pK19-*Δ1608*, pK19-*Δ1962*, pK19-*Δ2122*, pK19-*Δ2213*, pK19-*Δ2277*, pK19-*Δ2358*, pK19-*Δ2382*, pK19-*Δ2449*, pK19-*Δ2582*, pK19-*Δ2709*, and pK19-*Δ2952* were transformed into GAS355, followed by two-step homologous recombination. The deletions of the corresponding gene fragment were confirmed *via* colony PCR using primer sets P-C5/P-C6, P-C11/P-C12, P-C17/P-C18, P-C23/P-C24, P-C29/P-C30, P-C35/P-C36, P-C41/P-C42, P-C47/P-C48, P-C53/P-C54, P-C59/P-C60, P-C65/P-C66, P-C71/P-C72, P-C77/P-C78, P-C83/P-C84, P-C89/P-C90, P-C95/P-C96, P-C101/P-C102, P-C107/P-C108, P-C113/P-C114, and P-C119/P-C120, respectively. The resulting mutant strains were named HB-*Δ0186*, HB-*Δ0219*, HB-*Δ0313*, HB-*Δ0324*, HB-*Δ0503*, HB-*Δ0908*, HB-*Δ1213*, HB-*Δ1302*, HB-*Δ1459*, HB-*Δ1608*, HB-*Δ1962*, HB-*Δ2122*, HB-*Δ2213*, HB-*Δ2277*, HB-*Δ2358*, HB-*Δ2382*, HB-*Δ2449*, HB-*Δ2582*, HB-*Δ2709*, and HB-*Δ2952*, respectively **(** Supplementary Table S1 **)**.

### Strain Construction for Protocatechuic Aldehyde and Vanillin Production

To restore the *pobA* gene from the *pobA*-deleted 4-HBA–producing strain, APS963, pK19-*pobA* was transformed into APS963 and followed by two-step homologous recombination, yielding the MA183 strain **(** Table 1 **)**. Verification of *pobA* recovery was confirmed through PCR using P-pobA-CF and P-pobA-CR primers. To gain a strain harboring partial deletion of *pcaHG* and insertion of *ubiC* ^pr^, pK19-*ΔpcaHG*::P_*sod*_-*ubiC* ^pr^ was transformed into the mother strain, MA183, and finally the MA225 strain was obtained **(** Table 1 **)**. The deletion of the 0.82 kb *pcaHG* locus and insertion of 1 kb *ubiC* ^pr^ were confirmed by using primers P-pca-CF and P-pca-CR. The strain MA303 with partial deletion of the *vanAB* fragment was constructed by transformation of pK19-*ΔvanAB* into the mother strain, MA225 **(** Table 1 **)**. The partial deletion of the *vanAB* fragment was confirmed by PCR using primers P-van-CF and P-van-CR. The *NCgl0324*-deleted strain, PV-*Δ0324*, was constructed by the transformation of pK19-*Δ0324* into MA303 **(** Table 1 **)**.

### Expression and Purification of NCgl0324 Protein

*E. coli* BL21 (DE3) harboring pET-0324 was cultured at 37°C until OD_600nm_ reached 0.5 in 300 mL LB broth supplemented with 50 mg/L kanamycin and induced with 1 mM isopropyl β-D-1-thiogalactopyranoside. After cultivation at 18°C for 16 h, the cells were harvested by centrifugation at 5,478 x *g* for 5 min, washed with PBS buffer, and lysed by using Novagen BugBuster. After centrifugation at 25,155 x *g* for 1 h, the supernatant was loaded onto an affinity column containing Ni-NTA agarose gel (Bio-Works, Uppsala, Sweden) equilibrated with column buffer (20 mM Tris-HCl, 300 mM NaCl, and pH 7.4). The column was washed with the wash buffer (20 mM Tris-HCl, 300 mM NaCl, 10 mM imidazole, and pH 7.4), and then eluted with the elution buffer (20 mM Tris-HCl, 300 mM NaCl, 300 mM imidazole, and pH 7.4). The purified NCgl0324-His tag protein was dialyzed (20 mM potassium phosphate and pH 7.6) and concentrated using a spin column and stored at -72°C until use.

### Determination of NCgl0324 Enzyme Activity

The reaction mixture, 1 mL, for measuring aromatic aldehyde reductase activity of NCgl0324-His tag consisted of 0.1 M potassium phosphate (pH 7.6), 0.4 mM NADPH, and 5 mM aromatic aldehyde. Enzyme reaction was started by adding NADPH at 25°C, stopped by adding 5 μL of 2 N HCl to 200 μL, and neutralized by adding 5 μL of 2 N NaOH. The concentrations of 4-HB alcohol, PC alcohol, and vanillyl alcohol were measured using high-performance liquid chromatography (HPLC) at 280 nm, and then reductase activity for aromatic aldehydes, that is, 4-HB aldehyde, PC aldehyde, and vanillin was defined as the amount of enzyme required to produce micromoles of the corresponding aromatic alcohol per min per milligram of protein. The reaction mixture, 1 mL, for measuring aromatic alcohol dehydrogenase activity of NCgl0324-His tag consisted of 0.1 M potassium phosphate (pH 7.6), 0.4 mM NADP^+^, and 5 mM aromatic alcohol. The enzyme reaction was started by adding NADP^+^ at 25°C, stopped by adding 5 μL of 2 N HCl to 200 μL, and neutralized by adding 5 μL of 2 N NaOH. The concentrations of 4-HB aldehyde, PC aldehyde, and vanillin were measured using HPLC at 280 nm, and then dehydrogenase activity for aromatic alcohols, that is, 4-HB alcohol, PC alcohol, and vanillyl alcohol was defined as the amount of enzyme required to produce micromoles of corresponding aldehyde per min per milligram of protein.

### Culture Media and Conditions for *C. glutamicum*

*C. glutamicum* strains were routinely grown at 32°C in the Luria–Bertani (LB) medium (tryptone 10 g/L, yeast extract 5 g/L, and NaCl 10 g/L), supplemented with 50 mg/L kanamycin and/or 4.5 mg/L chloramphenicol (if necessary). To test the toxic effect of aromatic aldehydes on wild-type *C. glutamicum*, the CGXII medium with 40 g/L d-glucose and different ranges of 4-HB aldehyde, PC aldehyde, and vanillin were used (Eggeling and Bott, 2005). In order to produce aromatic aldehydes and alcohols in 250-mL baffled flasks, strains grown on LB agar plates for 36–48 h were suspended in saline and inoculated into 25 mL of the flask fermentation medium (Syukur Purwanto et al., 2018), followed by cultivation for 42–48 h with vigorous shaking at 240 rpm. Cell growth was measured by using the spectrophotometer (UV-2550, Shimadzu, Japan) and expressed as CDW (cell dry weight, g/L) by multiplying the measured OD_600nm_ by 0.25. When the color of the culture broth turned brown, the OD was measured after washing two to three times with distilled water. All the experiments were performed in triplicate, and mean and standard deviation were given.

### Analytical Procedures

The concentrations of 4-HBA, 4-HB aldehyde, 4-HB alcohol, PCA, PC aldehyde, PC alcohol, vanillate, vanillin, and vanillyl alcohol were determined using HPLC equipped with a UV detector and an Eclipse XDB-C18 (4.6 × 150 mm, 5 µm, Agilent) column. The analysis of 4-HBA, 4-HB aldehyde, and 4-HB alcohol from *C. glutamicum* was performed as previously described (Kim et al., 2020). For analysis of PCA, PC aldehyde, PC alcohol, vanillate, vanillin, and vanillyl alcohol, 1% acetic acid and acetonitrile (85:15, v/v) were used as a mobile phase at a flow rate of 0.8 mL/min, and metabolites were detected at 280 nm. To obtain crude extracts from *C. glutamicum*, the cells were disrupted with glass beads (BioSpec Products, Oklahoma, United States), and the supernatant was obtained by centrifugation at 11,400 x *g* for 30 min. Ten micrograms of denatured proteins were loaded on 10% gel in the SDS-PAGE (sodium dodecyl sulfate-polyacrylamide gel electrophoresis) experiment. The protein concentration was determined using the Pierce BCA protein assay kit (Thermo Fisher Scientific Inc., Massachusetts;, United States).

## Results

### Systematic Search for Candidate Genes Related to 4-Hydroxybenzaldehyde Reduction in *C. glutamicum*

Reduction of aromatic aldehydes can be catalyzed by various ADH/aldehyde reductase superfamilies. To date, functionally identified genes and enzymes involved in these reactions are little known in *C. glutamicum*. We preliminarily considered 19 candidate genes annotated as ADHs, oxidoreductases related to aryl–alcohol dehydrogenases, aldo–keto reductases, and short-chain dehydrogenases from bioinformatics analysis of *C. glutamicum* ATCC 13032 using UniProt and CoryneRegNet websites **(** Supplementary Table S5 **)**. In addition, six SDR (short-chain dehydrogenase/reductases) and five MDR (medium-chain dehydrogenase/reductase) families harboring the common cofactor binding site “TGXXXGXG” and the zinc-containing ADH signature “GHEX_2_GX_5_(G,A)X_2_(I,V,A,C,S),” respectively, were mined from the *C. glutamicum* genome database (de Smidt et al., 2008; Kavanagha et al., 2008; Knoll and Pleiss, 2008; Persson et al., 2009). Moreover, since deletion of *adhP*, *fucO*, *eutG*, *yjgB*, *yahK*, *ybbO*, *gldA*, *dkgA*, *yghA*, and/or *yqhD* in *E. coli* led to improved accumulation of several aldehydes (Rodriguez and Atsumi, 2012; Kunjapur et al., 2014; Rodriguez and Atsumi, 2014), BLAST search was performed by using these 10 proteins from *E. coli* as a query within the *C. glutamicum* genome. Ten proteins, that is, NCgl0219, NCgl0313, NCgl0324, NCgl0503, NCgl1003, NCgl1112, NCgl2053, NCgl2277, NCgl2709, and NCgl2952 were classified as candidates that showed a BLASTP expect value less than E^−20^. Likewise, three ADHs, that is, NCgl0219, NCgl0324, and NCgl2709 were chosen using *ADH6*-encoded ADH from *S. cerevisiae* as a query (Hansen et al., 2009). FudC encoded by *NCgl0324* from *C. glutamicum*, which was identified as a dehydrogenase with higher specificity toward furfural, was included (Tsuge et al., 2016). As some genes, such as *CGS9114*_*RS10340*, *CGS9114*_*RS09230*, *CGS9114*_*RS09375*, *CGS9114*_*RS11565*, *CGS9114*_*RS06005*, and *CGS9114*_*RS01115*, were significantly expressed in *C. glutamicum* S9114 in response to biotransformation of furaldehydes and benzaldehydes (Zhou et al., 2019), and the corresponding proteins within ATCC 13032, that is, NCgl0168, NCgl2277, NCgl0908, NCgl 1962, NCgl1608, and NCgl2709, respectively, were included in the screening. Of the 28 explored genes, *NCgl1112* was already deleted in the model GAS355 strain (Kim et al., 2020). Overall, 27 genes were systematically analyzed for identification of enzymes that reduce aromatic aldehydes to corresponding aromatic alcohols in *C. glutamicum* **(** Supplementary Table S5 **)**.

### Identification of Enzymes Responsible for Reduction of 4-Hydroxybenzaldehyde to 4-Hydroxybenzyl Alcohol in *C. glutamicum*

**FIGURE 2:**
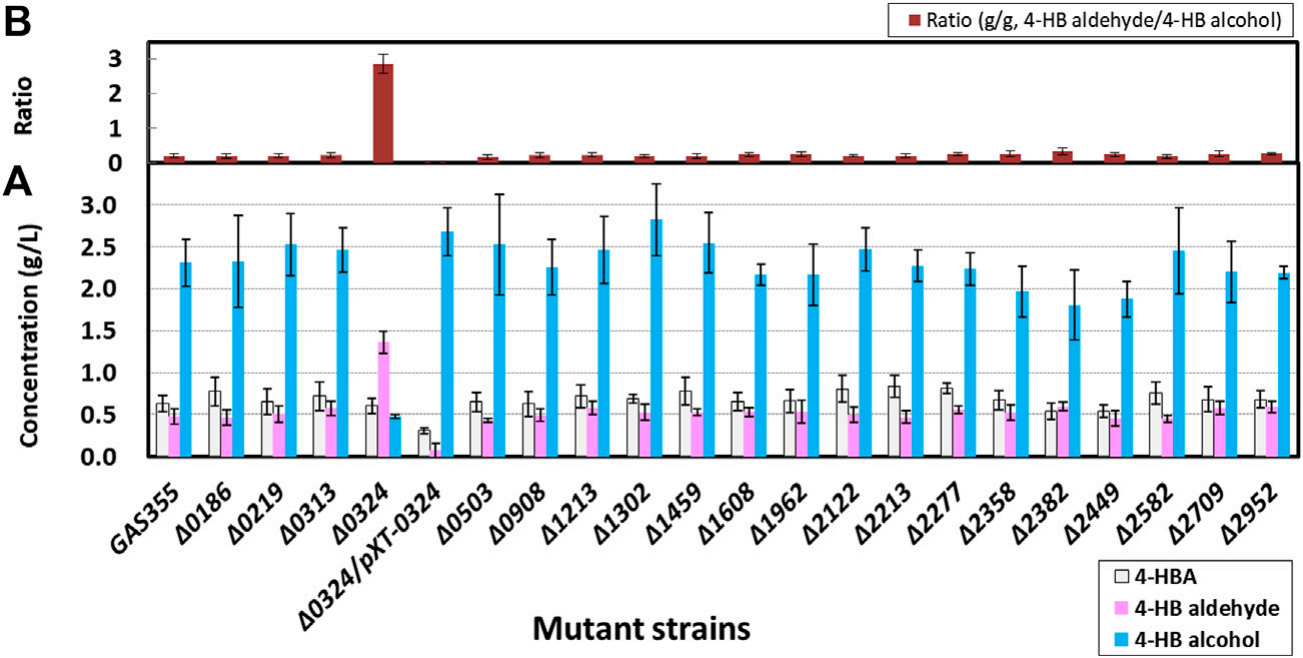
Production of 4-hydroxybenzoate, 4-hydroxybenzaldehyde, and 4-hydroxybenzyl alcohol. **(A)** and the ratio of 4-hydroxybenzaldehyde to 4-hydroxybenzyl alcohol **(B)** by 20 in-frame deletion *C. glutamicum* mutants responsible for 4-hydroxybenzyaldehyde reduction in baffled flasks. The cells were grown in flask fermentation medium for 42 h at 32°C with vigorous shaking at 240 rpm. Mean and standard deviation of triplicate cultivation are given.

With the aim of screening genes encoding aromatic alcohol dehydrogenases or aromatic aldehyde reductases (AARs), *C. glutamicum* GAS355 producing 4-HB alcohol was used as the model strain (Kim et al., 2020; Table 1 and Figure 1A). Upon transformation of GAS355 with the constructed knockout vectors and two-step homologous recombination, 20 gene deletion mutants among selected 27 candidates were successfully constructed, and the deletion of corresponding genes was verified through PCR analysis **(** Supplementary Figure S1 **)**. Using these mutants, production of 4-HB aldehyde and 4-HB alcohol and the ratio of 4-HB aldehyde to 4-HB alcohol were investigated in flask cultures with 80 g/L d-glucose **(** Figure 2 **)**. The parental strain GAS355 produced 2.31 ± 0.28 g/L of 4-HB alcohol as a main product and 0.47 ± 0.09 g/L of 4-HB aldehyde as a minor product, which means endogenous aldehyde reductase(s) readily converted 4-HB aldehyde into 4-HB alcohol. Nineteen mutants mainly produced 4-HB alcohol, but after 48 h of cultivation, the deletion mutants did not accumulate higher titers of 4-HB aldehyde than the control strain. By contrast, the *NCgl0324* deletion mutant, HB-*Δ0324*, accumulated 1.36 ± 0.09 g/L of 4-HB aldehyde as the dominant product (2.9-fold more than the parental strain) and 0.48 ± 0.03 g/L of 4-HB alcohol (4.8-fold less than the control). Thus, in the absence of the NCgl0324 enzyme, less 4-HB aldehyde was converted to 4-HB alcohol. Next, genetic complementation by plasmid-borne expression of *NCgl0324* (pXT-0324) in strain HB-*Δ0324* was analyzed to exclude effects by non-intentional secondary mutations. It was found that the 4-HB aldehyde titer in flask culture was decreased, while the 4-HB alcohol titer was increased, which indicated that the NCgl0324 enzyme complemented the deletion effect of *NCgl0324* **(** Figure 2 **)**. Accordingly, we identified *NCgl0324* as the primary gene responsible for conversion of 4-HB aldehyde to 4-HB alcohol in *C. glutamicum*.

### Catalytic Activity of NCgl0324 Toward Reduction of Aromatic Aldehydes

In the previous study, Tsuge et al. (2016) demonstrated that NCgl0324 (designated FudC) is an NADPH-dependent dehydrogenase that preferentially reduces furfural to furfuryl alcohol in *C. glutamicum*. Since it was unknown if NCgl0324 shows activity toward aromatic aldehydes, His-tagged NCgl0324 protein was purified from *E. coli* **(** Supplementary Figure S2 **)**, and its specific activity was determined using 4-HB aldehyde, PC aldehyde, or vanillin as a substrate with NADPH as a cofactor. The specific activity of NCgl0324 toward 4-HB aldehyde, PC aldehyde, and vanillin was 2.57 ± 0.14 µmol/min/mg, 3.15 ± 0.29 µmol/min/mg, and 4.59 ± 0.17 µmol/min/mg, respectively **(** Table 2 **)**, representing that NCgl0324 is characterized by aromatic aldehyde reductase (AAR) activity. To determine whether NCgl0324 expresses activity for the reverse oxidation reaction, its activity was measured using aromatic alcohols as substrates and NADP^+^ as a cofactor. The specific activities of NCgl0324 toward 4-HB alcohol, PC alcohol, and vanillyl alcohol were 0.50 ± 0.08 µmol/min/mg, 0.53 ± 0.10 µmol/min/mg, and 0.91 ± 0.06 µmol/min/mg, respectively **(** Table 2 **)**, which exhibit about 19.5, 16.8, and 19.8% activities compared to those toward the corresponding aromatic aldehyde. These results revealed that NCgl0324 has a higher activity for the reduction of aromatic aldehydes than for the oxidation of aromatic alcohols. Thus, our enzyme activity data indicate that NCgl0324 has a broad range of substrates and catalyzes the reduction of aromatic aldehydes to the corresponding aromatic alcohols, along with reduction activity toward furfural.

**TABLE 2:** Specific activity of the NCgl0324 enzyme for aromatic aldehydes and alcohols.

Enzyme Activity	Substrate^a^	μmol/min/mg Protein
Aromatic aldehyde reductase activity	4-hydroxybenzaldehyde	2.57 ± 0.14
	Protocatechuic aldehyde	3.15 ± 0.29
	Vanillin	4.59 ± 0.17
Aromatic alcohol dehydrogenase activity	4-hydroxybenzyl alcohol	0.50 ± 0.08
	Protocatechuic alcohol	0.53 ± 0.10
	Vanillyl alcohol	0.91 ± 0.06

a Substrate concentrations for determination of aromatic aldehyde reductase activity were 0.4 mM NADPH, 5 mM 4-hydroxybenzaldehyde, protocatechuic aldehyde, or vanillin. Substrate concentrations for determination of aromatic alcohol dehydrogenase activity were 0.4 mM NADP^+^ and 4-hydroxybenzyl alcohol, protocatechuic alcohol, or vanillyl alcohol. The data are averages from three independent measurements.

### Deletion of *NCgl0324* Improved Production of Protocatechuic Aldehyde by Engineered *C. glutamicum*

**FIGURE 3:**
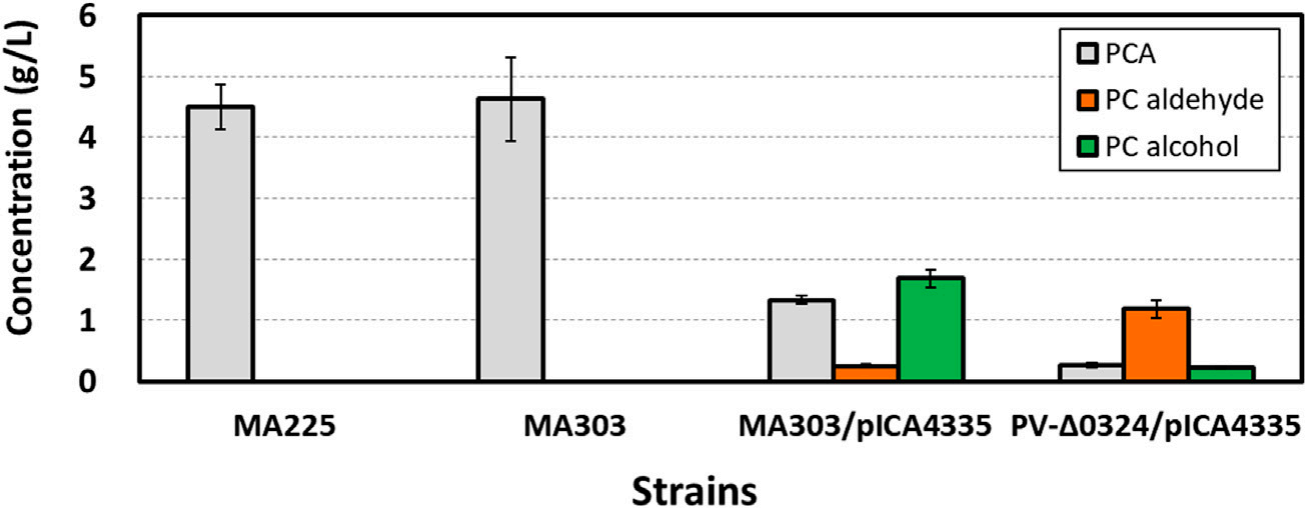
Effect of *NCgl0324* deletion on the production of protocatechuate, protocatechuic aldehyde, and protocatechuic alcohol by *C. glutamicum* mutants harboring the *car*-expressing pICA4335 plasmid in flask culture. Cells were grown in a flask fermentation medium for 42–48 h at 32°C with vigorous shaking at 240 rpm. Mean and standard deviation of triplicate cultivation are given.

The finding that NCgl0324 is active as an aromatic aldehyde reductase with PC aldehyde as the substrate prompted us to investigate whether *NCgl0324* knockout could block the conversion of this aromatic aldehyde to PC alcohol. Therefore, the PC aldehyde–producing strain was engineered from 4-HBA–producing APS963 (Kim et al., 2020; Figure 1B). First, hydroxylation of 4-HBA to PCA was engineered. Since the hydroxylase gene *pobA* was deleted in the 4-HBA–producing strain APS963, it was restored using pK19-*pobA* to yield the strain MA183 **(** Supplementary Figure S3A **)**. We reasoned that blockage of PCA degradation is required for PCA production as PCA is degraded to β-carboxymuconate by protocatechuate dioxygenase encoded by *pcaHG*. Using pK19*-ΔpcaHG*,*::*P_*sod*_ *-ubiC* ^pr^, the strain MA225 was constructed through a partial deletion of the *pcaHG* locus and simultaneous insertion of the mutated *ubiC*, which is needed to convert chorismate to 4-HBA **(** Supplementary Figure S3B **)**. The strain MA225 produced 4.5 ± 0.36 g/L of PCA with no accumulation of 4-HBA in flask culture after 42 h **(** Figure 3 **)**. In vanillate catabolism of *C. glutamicum*, the *vanAB* gene products, vanillate demethylase subunits A and B, convert vanillate to PCA (Merkens et al., 2005). Deletion of *vanAB* genes in MA225 using pK19*-ΔvanAB* yielded the strain MA303, which was also used as the base strain for production of the related aromatic aldehyde, vanillin **(** Supplementary Figure S3C **)**.

Next, we aimed to achieve PC aldehyde biosynthesis by reduction of PCA by a plasmid-borne expression of the heterologous *car* gene encoding the carboxylic acid reductase from *Nocardia iowensis* **(** Figure 1B **)**. The control strain MA303 produced 4.63 ± 0.69 g/L of PCA, whereas the strain carrying the plasmid pICA4335 for *car* expression produced 1.33 ± 0.07 g/L of PCA, 0.25 ± 0.03 g/L of PC aldehyde, and 1.69 ± 0.14 g/L of PC alcohol in flask cultures, but no detection of 4-HBA, 4-HB aldehyde, and 4-HB alcohol **(** Figure 3 **)** was observed. In spite of incomplete conversion of PCA into PC aldehyde and alcohol, it is evident that PC aldehyde was accumulated from PCA by the action of carboxylic acid reductase, followed by accumulation of a high titer of PC alcohol from PC aldehyde by the action of endogenous aldehyde reductase(s). Finally, in order to increase accumulation of PC aldehyde, a PV-*Δ0324* strain was constructed by deletion of *NCgl0324* from MA303 **(** Figure 1B; Table 1 **)**. In flask culture, the PV-*Δ0324* strain with pICA4335 enabled production of 1.18 ± 0.15 g/L of PC aldehyde and 0.22 ± 0.03 g/L of PC alcohol, which corresponded to a 4.8-fold increase of PC aldehyde and a 7.5-fold decrease of PC alcohol in comparison to the *NCgl0324*-positive control strain carrying pICA4335 **(** Figure 3 **)**. These results revealed that *NCgl0324* knockout not only benefitted 4-HB aldehyde production but also led to higher accumulation of PC aldehyde by the engineered *C. glutamicum* PV-*Δ0324*.

### Higher Accumulation of Vanillin in Engineered *C. glutamicum* by *NCgl0324* Knockout

**FIGURE 4:**
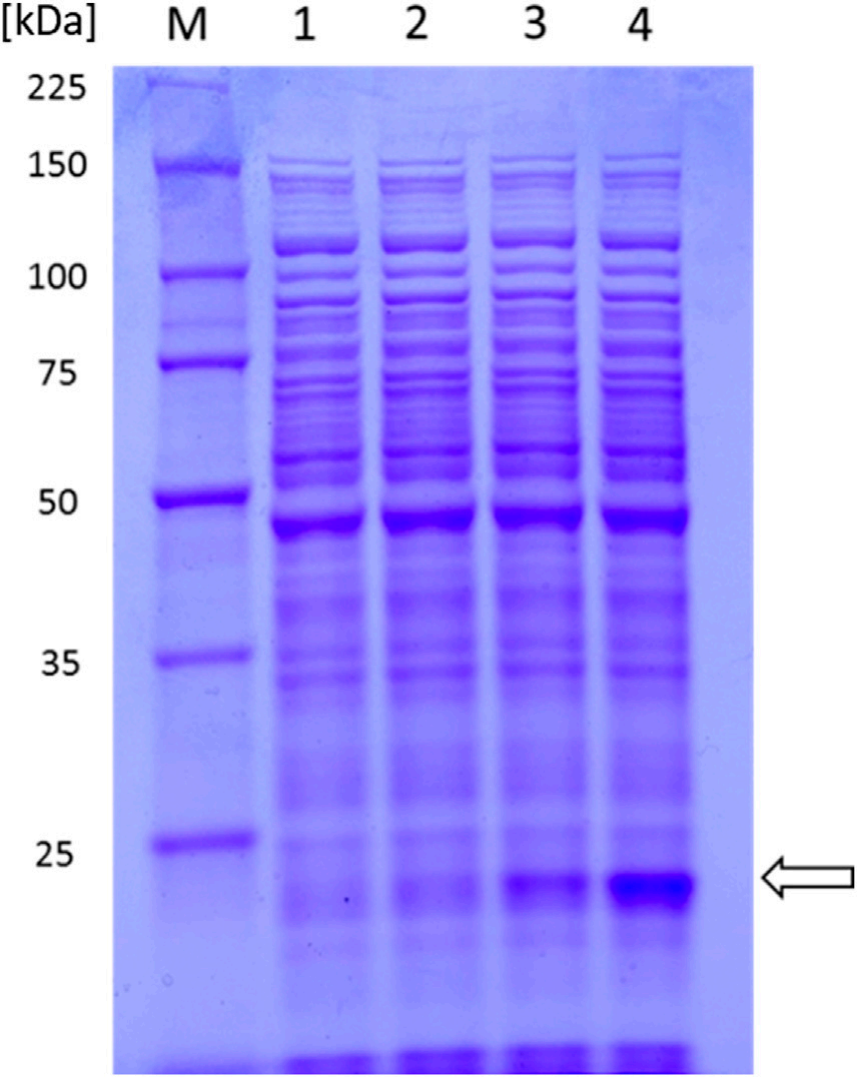
SDS-PAGE of *C. glutamicum* MA225 crude extracts carrying different vectors for expression analysis of the *comt* gene. 1. MA225; 2. MA225 with pYL230; 3. MA225 with pYL200; and 4. MA225 with pYL250.

After having shown the beneficial effect of *NCgl0324* knockout on the production of two aromatic aldehydes without the methoxy group, we attempted to produce another important aromatic aldehyde, vanillin, which possesses a methoxy group. To enable vanillin production, an artificial biosynthetic pathway expressing a mutated *comt* gene encoding murine catechol *O*-methyltransferase (COMT, Y200L) and the *car* gene, was introduced **(** Figure 1B **)**. Soluble COMT from *Rattus norvegicus* is known to catalyze *O*-methylation of PCA and PC aldehyde to yield vanillate and vanillin, respectively, using *S*-adenosyl-l-methionine (SAM) as a methyl donor. Moreover, enzyme activity and substrate specificity toward 3′-OH of PCA/PC aldehyde were enhanced by enzyme engineering of COMT, yielding the Y200L mutant (Law et al., 2016). Therefore, a codon-optimized mutant form of the *comt* ^m^ gene was synthesized and cloned into the expression vector pCXE50 **(** Supplementary Table S4 **)**. MA225 did not produce vanillate, while cells bearing the resulting pYL200 plasmid yielded 0.12 ± 0.01 g/L of vanillate, which demonstrated the functional expression of *comt* ^m^ from *R. norvegicus* in *C. glutamicum* **(** Table 3 **)**. To increase *comt* ^m^ expression and, as a result, vanillate production, we constructed plasmids pYL230 or pYL250 either by adding an extra sequence to TIR of the *tuf* promoter or by removing 0.18 kb fragment of the transcriptional terminator region from pYL200, respectively **(** Supplementary Figure S4 **)**. MA225-harboring pYL250 represented a noticeable production of COMT protein compared to cells with pYL200 or pYL230 **(** Figure 4 **)**. Consequently, a vanillate titer of 0.17 ± 0.02 g/L was obtained using MA225 with pYL250, which was 43% higher than that by MA225 with pYL200 **(** Table 3 **)**. It is evident that the deletion of an unnecessary fragment of the terminator yielded increased production of the COMT enzyme and vanillate titer. After having shown successful production of vanillate by the base strain MA225, pYL250 was used to transform the *vanAB*-deleted strain MA303 and the resultant strain produced 0.24 ± 0.03 g/L of vanillate. This indicated that the blockage of vanillate demethylation is significant for production of vanillate **(** Figure 1B **)**. Upon addition of 0.5 g/L l-methionine to the culture medium in order to increase the availability of SAM for methylation reaction, the highest vanillate product titer of 0.38 ± 0.04 g/L was produced.

**TABLE 3:** Production of vanillate using recombinant *C. glutamicum* strains with different plasmids for expression of the *comt* ^m^ gene in flask culture.

Strain	Plasmid	CDW*	PCA (g/L)**	Vanillate (g/L)	Condition
MA225	-	14.0 ± 0.35	4.99 ± 0.20	-	-
MA225	pYL200	15.6 ± 0.48	5.79 ± 0.34	0.12 ± 0.01	-
MA225	pYL230	15.7 ± 0.71	4.01 ± 0.70	0.05 ± 0.01	-
MA225	pYL250	15.7 ± 0.42	4.85 ± 0.50	0.17 ± 0.02	-
MA303	pYL250	15.1 ± 1.04	4.78 ± 0.27	0.24 ± 0.03	-
MA303	pYL250	15.1 ± 1.73	3.44 ± 0.67	0.38 ± 0.04	Adding 0.5 g/L L-methionine

*CDW, cell dry weight.

**PCA, protocatechuate.

**FIGURE 5:**
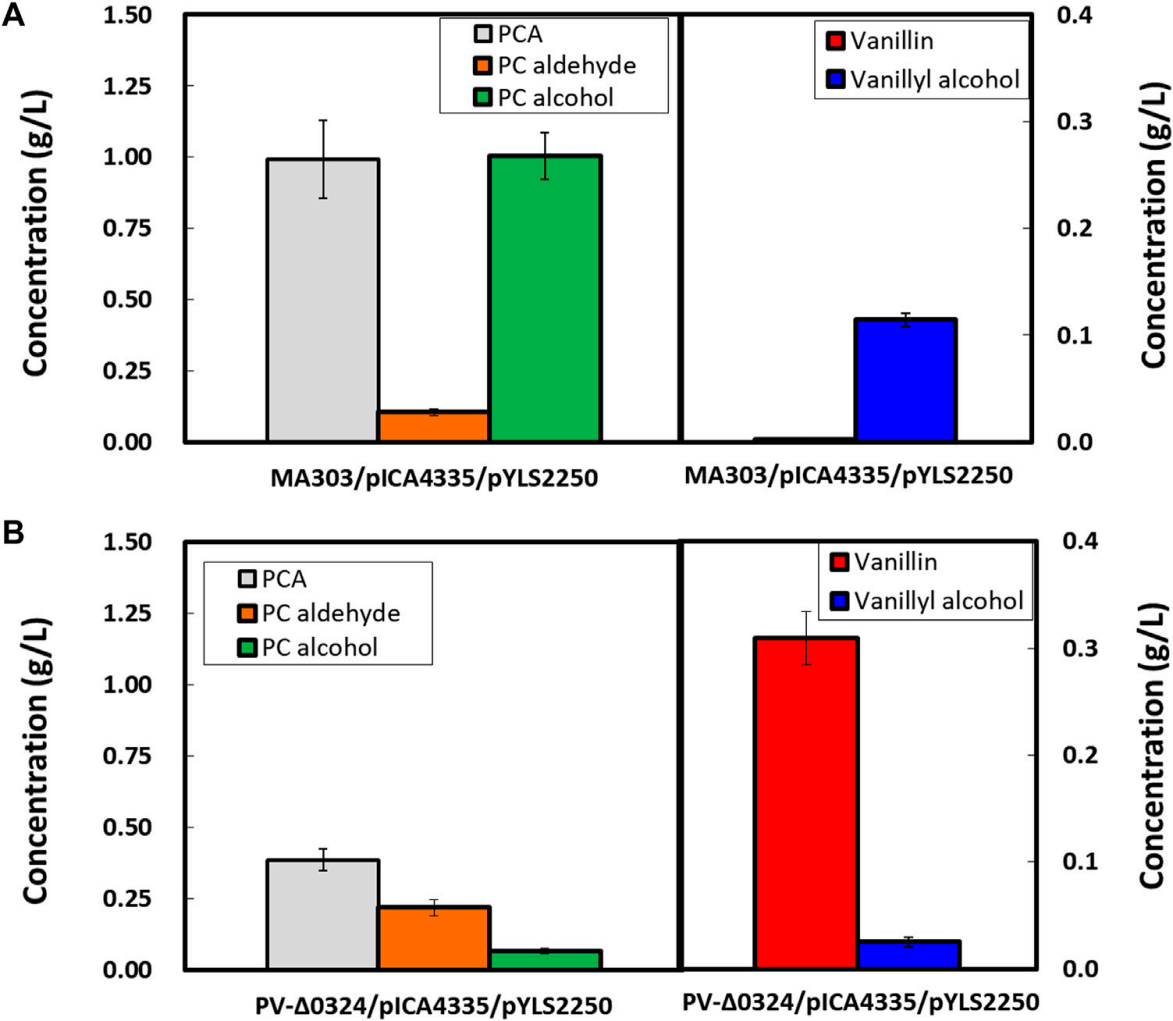
Effect of *NCgl0324* deletion on the production of vanillate, vanillin, vanillyl alcohol, protocatechuate, protocatechuic aldehyde, and protocatechuic alcohol by *C. glutamicum* mutants MA303 **(A)** and PV-*Δ0324* **(B)** harboring *car*-expressing pICA4335 and *comt* ^m^-expressing pYLS2250 plasmids in flask culture. The cells were grown in flask fermentation medium supplemented with 0.5 g/L L-methionine for 48 h at 32°C with vigorous shaking at 240 rpm. Mean and standard deviation of triplicate cultivation are given.

With the aim of vanillin and vanillyl alcohol production from PCA-producing strains, plasmids pICA4335 and pYLS2250 for the expressions of *car* and *comt* were used to transform strain MA303 and then cultivated for 48 h using 80 g/L d-glucose supplemented with 0.5 g/L l-methionine. If provisions of NADPH and SAM are efficient, PCA can be converted to various products by reaction sequences cascading CAR, COMT, and AARs, generating PC aldehyde (by CAR), PC alcohol (by CAR and AARs), vanillate (by COMT), vanillin (by CAR and COMT), or vanillyl alcohol (by CAR, COMT, and AARs). Consequently, 0.99 ± 0.14 g/L PCA, 0.11 ± 0.01 g/L PC aldehyde, 1.00 ± 0.08 g/L PC alcohol, and 0.11 ± 0.01 g/L vanillyl alcohol were produced after 48 h cultivation in baffled flasks **(** Figure 5A **)**. The finding that vanillin was not detected in the culture broth indicated that vanillin is rapidly reduced to vanillyl alcohol by endogenous AAR(s). Poor methylation of PCA and PC aldehyde may likely explain that the PC alcohol titer was 9.6-fold higher than the titer for vanillyl alcohol. When the *NCgl0324*-deleted strain PV-*Δ0324* was transformed with plasmids for co-expression of *car* and *comt* genes, the vanillin titer reached to 0.31 ± 0.03 g/L, while vanillyl alcohol was significantly decreased to 0.03 ± 0.00 g/L **(** Figure 5B **)**. Similarly, PC aldehyde was increased to 0.22 ± 0.03 g/L and PC alcohol was decreased to 0.07 ± 0.01 g/L. Therefore, we demonstrated that *NCgl0324* knockout led to higher production of vanillin by recombinant *C. glutamicum*.

### Growth and Production Profiles of Engineered *C. glutamicum* Strains in Flask Culture

**FIGURE 6:**
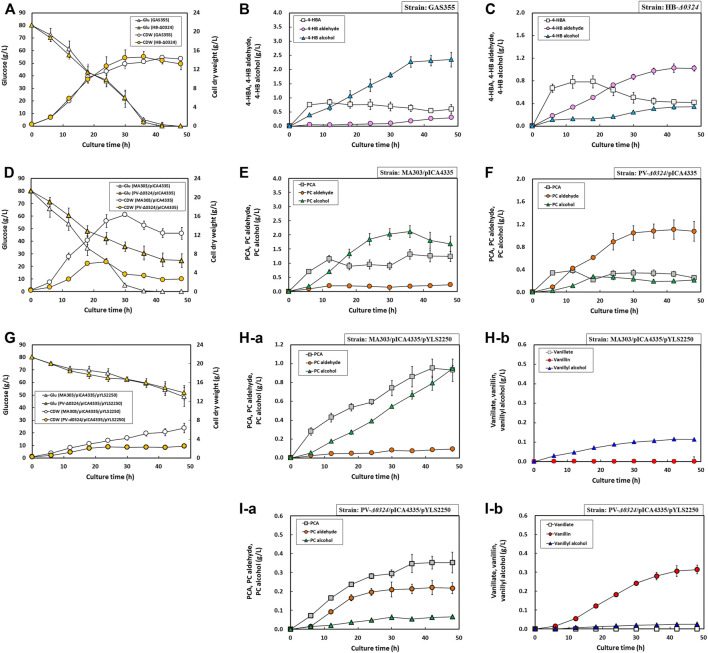
Growth and production profiles of *C. glutamicum* mutant strains for 48 h in flask culture. **(A)** Growth and glucose consumption profiles of GAS355 and HB-*Δ0324* strains for 48 h. **(B)** Production profiles of 4-HBA, 4-HB aldehyde, and 4-HB alcohol by GAS355 for 48 h. **(C)** Production profiles of 4-HBA, 4-HB aldehyde, and 4-HB alcohol by HB-*Δ0324* for 48 h. **(D)** Growth and glucose consumption profiles of strains MA303 and PV-*Δ0324* with plasmid pICA4335 for 48 h. **(E)** Production profiles of PCA, PC aldehyde, and PC alcohol by MA303 with pICA4335 for 48 h. **(F)** Production profiles of PCA, PC aldehyde, and PC alcohol by PV-*Δ0324* with pICA4335 for 48 h. **(G)** Growth and glucose consumption profiles of strains MA303 and PV-*Δ0324* with plasmids pICA4335 and pYLS2250 for 48 h **(H–a)** Production profiles of PCA, PC aldehyde, and PC alcohol by MA303 with pICA4335 and pYLS2250 for 48 h **(H–b)** Production profiles of vanillate, vanillin, and vanillyl alcohol by MA303 with pICA4335 and pYLS2250 for 48 h **(I–a)** Production profiles of PCA, PC aldehyde, and PC alcohol by PV-*Δ0324* with pICA4335 and pYLS2250 for 48 h **(I–b)** Production profiles of vanillate, vanillin, and vanillyl alcohol by PV-*Δ0324* with pICA4335 and pYLS2250 for 48 h. Most cells were grown in flask fermentation medium for 48 h at 32°C with vigorous shaking at 240 rpm. MA303 and PV-*Δ0324* with pICA4335 and pYLS2250, respectively, were cultivated in supplementary medium with 0.5 g/L L-methionine. Mean and standard deviation of triplicate cultivation are given.

Aromatic aldehydes are known to be detrimental to cell growth, which hampers their production (Zaldivar et al., 1999; Kunjapur et al., 2014). To monitor growth and production profiles of engineered *C. glutamicum* strains in response to biosynthesis of aromatic aldehydes and alcohols in more detail, flask cultivations were performed using minimal medium with 80 g/L d-glucose for 48 h. As expected, the cultivation of GAS355 showed that 4-HB alcohol production was steadily increased, reaching 2.35 ± 0.26 g/L after 48 h, but 4-HB aldehyde production did not exceed 0.30 ± 0.01 g/L due to the activity of endogenous AAR(s) **(** Figure 6B **)**. Conversely, HB-*Δ0324* displayed a gradual increase of 4-HB aldehyde production (1.02 ± 0.05 g/L) but a sharp decrease of 4-HB alcohol production (0.34 ± 0.02 g/L) as a consequence of *NCgl0324* gene deletion **(** Figure 6C **)**. Although the production of more toxic 4HB aldehyde in HB-*Δ0324* was 3-fold higher than that in GAS355, the strains GAS355 and HB-Δ0324 showed comparable biomass and d-glucose consumption profiles with specific growth rates of 0.28 ± 0.01 /h and 0.27 ± 0.00 /h, respectively **(** Figure 6A **)**.

When MA303 harboring pICA4335 (designated MA-I303 strain) was cultured in baffled flasks, the biomass (cell dry weight, CDW) was rapidly increased up to a maximum of 16.31 ± 0.43 g/L at 30 h with a specific growth rate of 0.29 ± 0.01 /h and then declined to 12.36 ± 1.26 g/L after 48 h **(** Figure 6D **)**. By contrast, the biomass of PV-*Δ0324* harboring pICA4335 (designated PV-I*Δ0324* strain) was slowly increased to a maximum of 6.30 ± 0.43 g/L at 24 h and declined to 2.7 ± 0.33 g/L after 48 h. The specific growth rate of cells was 0.22 ± 0.01 /h, which was 33% slower than that of MA-I303. The added 80 g/L d-glucose was completely consumed during the 36-h cultivation in MA-I303, whereas 25 g/L d-glucose remained in PV-I*Δ0324* during 48 h. PC alcohol and PC aldehyde were accumulated as major products in strains MA-I303 and PV-I*Δ0324*, respectively. In MA-I303, PC alcohol was rapidly accumulated as a major product (2.12 ± 0.21 g/L) until 36 h and then decreased to 1.69 ± 0.27 g/L after complete depletion of d-glucose **(** Figure 6E **)**. Similar to cultivation of HB-*Δ0324*, the PC aldehyde titer in PV-I*Δ0324* was gradually increased to 1.07 ± 0.18 g/L over 48 h, whereas PC alcohol production was 7.9-fold lower than the production in MA-I303 **(** Figure 6F **)**. Overall, compared to the MA-I303 strain, slower cell growth and d-glucose consumption in PV-I*Δ0324* were probably associated with a higher accumulation of the more toxic PC aldehyde.

The production performance of MA303 and PV-*Δ0324* strains harboring pICA4335 and pYLS2250 was also evaluated in flasks culture supplemented with 0.5 g/L l-methionine. Cell growth and d-glucose consumption of both strains were significantly delayed during cultures compared to those of 4-HB aldehyde/alcohol or PC-aldehyde/alcohol producing strains **(** Figure 6G **)**. Cultivation of MA303-harboring pICA4335 and pYLS2250 (designated MA-IY303 strain) reduced the specific growth rate (0.208 ± 0.01 /h) and final biomass (6.35 ± 1.01 g/L) during 48 h. In addition, cultivation of PV-*Δ0324* harboring pICA4335 and pYLS2250 (designated PV-IY*Δ0324* strain) led to significant reduction of the specific growth rate (0.14 ± 0.01 /h) and final biomass (2.55 ± 0.47 g/L) during the 48-h cultivation. At the same time, d-glucose consumption of both strains was also severely retarded by accumulation of various aromatic aldehydes and alcohols, including vanillyl alcohol and vanillin **(** Figure 6G **)**. Even though the accumulated PC alcohol titer (0.95 ± 0.03 g/L) in the MA-IY303 strain was 55% reduced in comparison to that in MA-I303 **(** Figure 6H **)**, d-glucose consumption and cell growth were remarkably impeded, which might be caused by accumulation of toxic vanillyl alcohol (0.11 ± 0.01 g/L). Cultivation of PV-IY*Δ0324* displayed production of 0.31 ± 0.02 g/L vanillin, 0.03 ± 0.00 g/L vanillyl alcohol, and 0.22 ± 0.03 g/L PC aldehyde (Figure 6I) and the lowest specific growth rate among tested strains, implying the highest toxicity of vanillin toward *C. glutamicum* among accumulated aromatic aldehydes and alcohols.

## Discussion

In recent years, efforts on production of industrially relevant aldehydes have shifted from plant extraction or chemical synthesis to direct fermentation using microbes engineered through systems metabolic engineering (Hayden, 2014; Kunjapur and Prather, 2015). However, accumulation of aldehydes in the microbial hosts poses a challenge due to its rapid and endogenous conversion to alcohols. In this study, 27 proteins were systematically selected based on literature search and bioinformatics analysis **(** Supplementary Table S5 **)** and a comprehensive *in vivo* screening was performed using the model strain, the 4-HB alcohol-producing *C. glutamicum*. Due to enzyme promiscuity with broad substrate spectrum (Tseliou et al., 2021), we hypothesized that each knockout of the 20 genes in GAS355 would lead to selection of several promising genes responsible for reduction of 4-HB aldehyde to 4-HB alcohol. Surprisingly, only the *NCgl0324* knockout mutant accumulated 2.9-fold more 4-HB aldehyde and 4.8-fold less 4-HB alcohol than the control strain, indicating that the *NCgl0324* gene product plays a pivotal role in the conversion of 4-HB aldehyde to 4-HB alcohol. Likewise, deletion of *NCgl0324* from MA303 led to 4.8-fold increase of PC aldehyde and a 7.5-fold decrease of PC alcohol, and similarly, to a remarkable increase of vanillin and 3.6-fold decrease of vanillyl alcohol. This implies that *NCgl0324* knockout enables direct production of structurally similar aromatic aldehydes such as 4-HB aldehyde, PC aldehyde, and vanillin from d-glucose in metabolically engineered *C. glutamicum* strains. Moreover, we suggest it can boost production of aromatic aldehydes and alcohols with structural diversity in *C. glutamicum*.

Previously, Tsuge et al. (2016) characterized that NCgl0324 from *C. glutamicum* is an NADPH-dependent ADH responsible for reduction of 5-membered heterocycle furfural to furfuryl alcohol and has a narrow substrate range in comparison to YqhD from *E. coli* (Jarboe, 2011). Thus, it needs to investigate whether NCgl0324 is responsible for reduction of structurally similar aromatic aldehydes to alcohols. Moreover, microbial engineering for aromatic aldehyde production has been executed in yeasts and *E. coli*. Among the 29 candidate proteins tested in *S. pombe*, inactivation of the *ADH6*-encoded ADH resulted in a 50% decrease in converting vanillin to vanillyl alcohol and finally yielded 45 mg/L vanillin from d-glucose (Hansen et al., 2009). In the engineered *E. coli* RARE strain, Kunjapur et al. (2014) observed that deletion of four genes, including *dkgA*, *yqhD*, *yahK*, and *yjgB*, is indispensable for production of benzaldehyde and vanillin. In particular, the RARE strain in combination with the introduction of the vanillin biosynthetic pathway produced 119 mg/L of vanillin from d-glucose. In addition, ADH6, YqhD, and DkgA were known to possess furfural reductase activity such as NCgl0324 but have a broad substrate spectrum toward medium-chain substrates (Larroy et al., 2002; Miller et al., 2009). Taking into consideration, BLAST analysis was performed using the experimentally proven five enzymes as a query within *C. glutamicum*. Proteins with high sequence similarity comprised three MDR family enzymes (NCgl0324, NCgl0219, and NCgl2709) and three homologs of 2,5-diketo-d-gluconic acid reductase (NCgl2277, NCgl1308, and NCgl0503) but no homologs with DkgA and YqhD. When 27 candidates were systematically mined based on the aforementioned rationale, we found only NCgl0324 is contributed to the conversion of aromatic aldehydes to aromatic alcohols, but the reverse reaction is not favored under physiological conditions of *C. glutamicum*. We also demonstrated that NCgl0324 has aromatic aldehyde reductase activity toward 4-HB aldehyde, PC aldehyde, and vanillin together with furfural, but a detailed enzyme characterization is required. Other gene products have presumably different catalytic activities or functions in redox reactions. For instance, *NCgl2709* encoding an alcohol dehydrogenase has been shown to be essential for growth on either ethanol or *n*-propanol, and chromosomal inactivation of the said gene rendered the strain incapable of growth on these two alcohols (Kotrbova-Kozak et al., 2007). 2,5-diketo-d-gluconic acid (2,5-DKG) reductase encoded by *NCgl2277* from *C. glutamicum* sharing 64% sequence similarity with DkgA from *E. coli* is an NADPH-dependent aldo–keto reductase that catalyzes the reduction of 2,5-DKG to 2-keto-l-gulonic acid (Kaswurm et al., 2012). The effect of *NCg0324* deletion is less obvious for 4-HB aldehyde production than for PC aldehyde production, while the catalytic activity of the NCgl0324 enzyme toward these two aldehydes is similar. One possibility is the presence of additional enzymes for converting 4-HB aldehyde to 4-HB alcohol. In this regard, the deletion of the single gene (other than *NCgl0324*) might have a minor influence due to the redundancy of these enzymes. To examine the additive effect, it might be helpful in evaluating the deletion effect of *NCgl2382* (Figure 2B) contributing and other genes, whose deletion might be contributing to the reduction of alcohols in the knockout strain of HB-*Δ0324*. Taken together, although screened many enzymes with a presumed broad spectrum of substrates are expected to provide potential aromatic aldehyde reductase activity, *in vivo* and *in vitro* results demonstrated that only NCgl0324 is crucial for metabolism of aromatic aldehyde reduction.

The GAS355 and HB-*Δ0324* harboring *car* gene in the genome produced 0.63 g/L and 0.6 g/L of 4-HBA, respectively, along with accumulation of 4-HB alcohol and 4-HB aldehyde. Likewise, MA303/pICA4335 and PV-*Δ0324*/pICA4335 harboring *car* gene in the plasmid produced 1.33 g/L and 0.26 g/L of PCA, respectively, along with production of PC aldehyde and PC alcohol. Accumulation of unwanted 4-HBA and PCA in engineered strains may be due to a poor expression of the *car* gene and/or a poor enzymatic activity of CAR in *C. glutamicum*. As the production of CAR protein was detected by SDS-PAGE in the previous study (Kim et al., 2020), a poor expression of the *car* gene in *C. glutamicum* was excluded. Another possibility is oxidation of the accumulated 4-HB aldehyde or PC aldehyde into 4-HBA or PCA by endogenous (aromatic) aldehyde dehydrogenase(s). Generally, aldehydes are metabolized into less toxic reduced alcohols or oxidized acids by diverse oxidoreductases in microbes (Shaw et al., 1992; Liu and Moon, 2009; Zhou et al., 2019). When *C. glutamicum* S911 was treated with toxic furaldehyde or benzaldehydes, 10 genes encoding putative aldehyde dehydrogenases and 27 genes encoding putative alcohol dehydrogenases were significantly upregulated (Zhou et al., 2019). In these perspectives, to decrease aromatic acids and increase aromatic aldehydes in the next study, we consider it a requisite to increase *car* expression and/or screen and delete gene(s) encoding aromatic aldehyde dehydrogenase(s). On the one hand, the MA-IY303 and PV-IY*Δ0324* strains did not produce vanillate, despite accumulation of 0.39–1.0 g/L PCA. This is likely due to deletion of *NCgl2578* encoding vanillin dehydrogenase in GAS355, MA303, and derived strains (Ding et al., 2015). While this enzyme has the highest activity on vanillin, it shows low activity on a wide range of substrates, such as 4-HB aldehyde and PC aldehyde. Even though *vdh*-deleted strains were used in this study, moderate or marginal levels of aromatic acids were produced, which proposes that other enzymes responsible for oxidation of 4-HB aldehyde or PC aldehyde to 4-HBA or PCA are still present in *C. glutamicum*.

Due to their electrophilic reactivity (LoPachin and Gavin, 2014), aldehydes show toxic effects in microbes, for example, inhibition of cell growth, cell lysis (Priefert et al., 2001), morphological changes (Huang et al., 2011), or formation of protein-DNA crosslinking (Chen et al., 2020). In this sense, one of the key issues in microbial aldehyde production is aldehyde toxicity to host strains (Kunjapur and Prather, 2015). As can be seen in Figure 6, *NCgl0324*-deleted strains producing PC aldehdye and/or vanillin exhibited relatively slower cell growth and d-glucose consumption than mother strains during cultures. Especially, vanillin production displayed the highest toxicity to *C. glutamicum* in comparison to PC aldehyde production. In contrast, although production of toxic 4-HB aldehyde in HB-*Δ0324* was 3-fold higher than that in the mother strain, both strains showed comparable cell mass and d-glucose consumption. To compare the response of wild-type *C. glutamicum* to externally added aromatic aldehydes, we measured half-maximal inhibitory concentrations (*K*i) on *C. glutamicum* ATCC 13032. The *K*i values of 4-HB aldehyde, PC aldehyde, and vanillin were 1.1 g/L, 0.72 g/L, and 1.3 g/L, respectively (Supplementary Figure S5), which indicated that PC aldehyde exhibits the highest toxicity among tested aldehydes against wild-type *C. glutamicum*. It is assumed that the conflicting toxic effect of aromatic aldehydes between engineered strains and wild-type resulted from intracellular accumulation and external supplementation of these aldehydes and from the difference in the genotype of each strain. Zaldivar et al. (1999) examined the effect of several aldehydes on *E. coli* growth, showing that 4-HB aldehyde is more toxic than vanillin, followed by syringaldehyde, furfural, and 5-hydroxymethyl furfural. When the toxic effects of these aldehydes were investigated in terms of cell growth and sugar consumption on the yeast *Trichosporon fermentans*, vanillin is more toxic than 4-HB aldehyde (Huang et al., 2011). Thus, the degree of toxicity to microbes seems to vary depending on the inherent characteristics of host strains. Some studies on vanillin have been reported to reduce or cope vanillin toxicity, for example, screening of vanillin-resistant mutants using chemical mutagenesis (Yoon et al., 2007), removal of vanillin using adsorbent resin (Hua et al., 2007), and production of less toxic glucovanillin *via* glucosylation of vanillin (Hansen et al., 2009). On the one hand, adaptive laboratory evolution (ALE) has been used to resolve chemical toxicity to microbes, for example, indole (Walter et al., 2020) or methanol (Hennig et al., 2020) in *C. glutamicum* and formaldehyde in *E. coli* (Chen et al., 2020). Perhaps, ALE is one way to evolve *C. glutamicum* strains with higher tolerance to aromatic aldehydes. Since vanillin production is significantly enhanced by adding l-methionine, it appears to be a plausible alternative to decouple vanillin production from cell growth through bioprocess optimization. Hitherto, a challenge posed to improve aromatic aldehyde production by overcoming aldehyde toxicity has to be addressed.

As vanillin is one of the most important aromatic aldehydes, *de novo* synthesis of vanillin by metabolically engineered microorganisms using renewable biomass is considered a very attractive bio-vanillin production technology. To this end, we first tried to construct vanillate-producing *C. glutamicum* strains by *comt* ^m^ overexpression through engineering of TIR or transcription terminator. The distance between the ribosome-binding site (RBS) and start codon is one of the factor influencing translation efficiency of protein and depends on promoter types (Kang et al., 2014; Lee, 2014). Since the distances in *tuf*, *sod*, *tac,* and *ilvC* promoters are 7, 8, 10, and 11 nucleotides, respectively, two nucleotides (nt) were added after the RBS of the *tuf* promoter in plasmid pYL200 (Lee, 2014). However, COMT production was severely decreased in MA225-harboring pYL230 compared to that in the control strain, revealing that increasing the distance between the RBS and start codon from 7 to 9 nt resulted in a negative effect on the translation efficiency of COMT protein. On the other hand, COMT production was remarkably enhanced when we removed about 0.18 kb region between the stop codon and terminator 1 structure in pYL200 according to secondary structure analysis of the *rrnB* transcription terminator (Supplementary Figure S4). Thus, removal of unnecessary fragment in front of the transcription terminator is beneficial for the overexpression of *comt* ^m^ in pYL250. Moreover, translation initiation rates and total ΔG of the *comt* ^m^ gene in the plasmids pYL200, pYL230, and pYL250 were determined using an RBS calculator in the Salis Lab (Salis, 2011; Supplementary Table S6). The result was in accordance with what we observed *in vivo*. The *comt* ^m^ gene has the highest translation initiation rate in pYL250, followed by pYL200 and last by pYL230. In addition, the mRNA stability of *comt* ^m^, as depicted by ΔG total, is highest in pYL250. Hence, we believed that the pYL250 is, indeed, the best construct in terms of both the increased translation initiation rate and mRNA stability. Moreover, inactivation of aromatic aldehyde reductase, NCgl0324, in combination with an extending vanillin biosynthetic pathway from 4-HBA in engineered *C. glutamicum* enabled production of 0.31 g/L of vanillin from d-glucose in flask culture. However, there are still high titers of by-products such as PCA and PC aldehyde that are not converted to methoxylated compounds. When l-methionine was added to the culture medium using MA303/pYL250, the vanillate titer increased by 58% **(** Table 3 **)**, which represented the importance of SAM pool required for methylation reaction. We, thus, expect to develop superior *C. glutamicum* strains for viable production of vanillin through further metabolic engineering, for example, the l-methionine biosynthetic pathway and regeneration of 5-methyltetrafolate and SAM pools.

## Conclusion

We demonstrated that *NCgl0324* knockout in artificial biosynthetic pathways for biosynthesis of aromatic aldehydes enables not only the development of *C. glutamicum* strains for producing 4-HB aldehyde, PC aldehyde, and vanillin but also can be applied to develop a platform strain for production of value-added versatile aromatic aldehydes. To the best of our knowledge, the titers of 4-HB aldehyde, PC aldehyde, and vanillin in this study are among the highest based on direct fermentation of microbes from simple carbon sources.

## Data Availability

The original contributions presented in the study are included in the article/Supplementary Material, further inquiries can be directed to the corresponding author.
