# Supplementary material for: Engineered Corynebacterium glutamicum as the Platform for the Production of Aromatic Aldehydes
Source: Front Bioeng Biotechnol. 2022 May 12;10:880277. doi: 10.3389/fbioe.2022.880277 (PMC9133326; doi:10.3389/fbioe.2022.880277)

**Supplementary Material**

**Engineered *Corynebacterium glutamicum* as the Platform for the Production of Aromatic Aldehydes**

***Hyun-Song Kim^1^, Jung-A Choi^1^, Bu-Yeon Kim^1,^*^†^*, Lenny Ferrer^2^, Jung-Min Choi^1^, Volker F. Wendisch^2^, Jin-Ho Lee^1^*^*^**

*^1^Department of Food Science & Biotechnology, Kyungsung University, Busan, Republic of Korea*

*^2^Genetics of Prokaryotes, Faculty of Biology and Center for Biotechnology, Bielefeld University, Bielefeld, Germany*

**^*^Corresponding author**

Tel: +82-51-663-4716, Fax: +82-504-238-7072

E-mail: [jhlee83@ks.ac.kr](mailto:jhlee83@ks.ac.kr)

**^†^Present address:** Genofocus Inc., Daejeon, Republic of Korea

**Supplementary Material**

**Supplementary Figure 1.** Verification of deletion of twenty candidate genes responsible for 4-hydroxybenzaldehyde reduction in *C. glutamicum* by PCR analysis. “C” and “M” refer to strain GAS355 and the corresponding mutant strain, respectively. Corresponding deletion mutant, PCR using primer set for confirmation of deletion of corresponding gene fragment, and deletion size are as follows.

(**A**) M1, HB-*Δ0186*; P-C5/P-C6; 0.3 kb. (**B**) M2, HB-*Δ0219*; P-C11/P-C12; 0.3 kb. (**C**) M3, HB-*Δ0313*; P-C17/P-C18; 0.3 kb. (**D**) M4, HB-*Δ0324*; P-C23/P-C24; 0.3 kb. (**E**) M5, HB-*Δ1213*; P-C41/P-C42; 0.3 kb. (**F**) M6, HB-*Δ0503*; P-C29/P-C30; 0.3 kb. (**G**) M7, HB-*Δ1302*; P-C47/P-C48; 0.3 kb. (**H**) M8, HB-*Δ1459*; P-C53/P-C54; 0.4 kb. (**I**) M9, HB-*Δ0908*; P-C35/P-C36; 1.14 kb. (**J**) M10, HB-*Δ1608*; P-C59/P-C60; 0.81 kb. (**K**) M11, HB-*Δ1962*; P-C65/P-C66; 0.72 kb. (**L**) M12, HB-*Δ2213*; P-C77/P-C78; 0.31 kb. (**M**) M13, HB-*Δ2277*; P-C83/P-C84; 0.3 kb. (**N**) M14, HB-*Δ2122*; P-C71/P-C72; 0.3 kb. (**O**) M15, HB-*Δ2358*; P-C89/P-C90; 0.3 kb. (**P**) M16, HB-*Δ2382*; P-C95/P-C96; 0.3 kb. (**Q**) M17, HB-*Δ2449*; P-C101/P-C102; 0.3 kb. (**R**) M18, HB-*Δ2582*; P-C107/P-C108; 0.3 kb. (**S**) M19, HB-*Δ2709*; P-C113/P-C114; 0.3 kb. (**T**) M20, HB-*Δ2952*; P-C119/P-C120; 0.3 kb.

**Supplementary Figure 2.** SDS-PAGE of purified NCgl0324 protein. NCgl0324 protein (lane 1) was purified from crude extract of *E. coli* BL21 (DE3) harboring pET-NCgl0324 by using affinity column containing Ni-NTA agarose gel. Arrows showed the purified NCgl0324 protein band.

**Supplementary Figure 3.** Verification of the constructed mutant strains MA183 (**A**), MA225 (**B**), and MA303 (**C**) by PCR analysis. (**A**) Confirmation of *pobA* recovery in MA183. PCR was conducted by using primers P-pobA-CF and P-pobA-CR. Lane 1 is PCR product of APS963 chromosome, showing 1.34 kb PCR band; lane 2 is PCR product of mutant MA183 chromosome, showing 2.53 kb PCR band by insertion of *pobA* gene. (**B**) Confirmation of *pcaHG* partial deletion and simultaneous *ubic*^pr^ insertion in MA225. PCR was conducted by using primers P-pca-CF and P-pca-CR. Lane 1 is PCR product of MA183 chromosome, showing 1.72 kb PCR band; lane 2 is PCR product of mutant MA225 chromosome, showing 1.94 kb PCR band. (**C**) Confirmation of *vanAB* partial deletion in MA303. PCR was conducted by using primers P-van-CF and P-van-CR. Lane 1 is PCR product of MA225 chromosome, showing 2.5 kb band; lane 2 is PCR product of MA303 chromosome, showing 1.2 kb PCR band.

**Supplementary Figure 4.** Comparison of DNA sequence in translation initiation region (TIR) of *tuf* promoter (**A**) and the secondary structure of *rrnB* transcription terminator (**B**) of constructed vectors, pYL200, pYL230, and pYL250. (**A**) The TIR of *tuf* promoter contains ribosome binding site (RBS) and *Eco*RI site in pYL200 and pYL230. Additional nucleotide sequence located between TIR and *Eco*RI site in pYL230. (**B**) The secondary structure of *rrnB* transcription terminator of pYL200 and pYL250. T1 and T2 mean the terminator 1 and 2; IR1 and IR2 mean the inverted repeat 1 and 2.

**Supplementary Figure 5.** Response of wild-type *C. glutamicum* to externally added 4-hydroxybenzylaldehyde (4-HB aldehyde), protocatechuic (PC) aldehyde, and vanillin. To determine the *K*i for 4-HB aldehyde, PC aldehyde, and vanillin, CGXII minimal medium with 40 g/L d-glucose and different concentrations of aromatic aldehydes from 0 to 1.5 g/L was used. A linear regression was performed to determine the half-maximal inhibitory concentrations (*K*i) on *C. glutamicum* ATCC 13032.

**Supplementary Table 1.** Bacterial strains and plasmids used to screen for aromatic aldehyde reduction in this study.

| Strain or  Plasmid | Characteristics | | Source |
| --- | --- | --- | --- |
| *Corynebacterium glutamicum* mutants | | |  |
| HB-*Δ0186*  HB-*Δ0219*  HB-*Δ0313*  HB-*Δ0324*  HB-*Δ0503*  HB-*Δ0908*  HB-*Δ1213*  HB-*Δ1302*  HB-*Δ1459*  HB-*Δ1608*  HB-*Δ1962*  HB-*Δ2122*  HB-*Δ2213*  HB-*Δ2277*  HB-*Δ2358*  HB-*Δ2382*  HB-*Δ2449*  HB-*Δ2582*  HB-*Δ2709*  HB-*Δ2952*  Plasmids  pK19-*Δ0186*  pK19-*Δ0219*  pK19-*Δ0313*  pK19-*Δ0324*  pK19-*Δ0503*  pK19-*Δ0908*  pK19-*Δ1213*  pK19-*Δ1302*  pK19-*Δ1459*  pK19-*Δ1608*  pK19-*Δ1962*  pK19-*Δ2122*  pK19-*Δ2213*  pK19-*Δ2277*  pK19-*Δ2358*  pK19-*Δ2382*  pK19-*Δ2449*  pK19-*Δ2582*  pK19-*Δ2709*  pK19-*Δ2952* | | GAS355 *ΔNCgl0186*; 0.3 kb fragment deletion within *NCgl0186*  GAS355 *ΔNCgl0219*; 0.3 kb fragment deletion within *NCgl0219*  GAS355 *ΔNCgl0313*; 0.3 kb fragment deletion within *NCgl0313*  GAS355 *ΔNCgl0324*; 0.3 kb fragment deletion within *NCgl0324*  GAS355 *ΔNCgl0503*; 0.3 kb fragment deletion within *NCgl0503*  GAS355 *ΔNCgl0908*; 1.14 kb fragment deletion within *NCgl0908*  GAS355 *ΔNCgl1213*; 0.3 kb fragment deletion within *NCgl1213*  GAS355 *ΔNCgl1302*; 0.3 kb fragment deletion within *NCgl1302*  GAS355 *ΔNCgl1459*; 0.4 kb fragment deletion within *NCgl1459*  GAS355 *ΔNCgl1608*; 0.81 kb fragment deletion within *NCgl1608*  GAS355 *ΔNCgl1962*; 0.72 kb fragment deletion within *NCgl1962*  GAS355 *ΔNCgl2122*; 0.3 kb fragment deletion within *NCgl2122*  GAS355 *ΔNCgl2213*; 0.31 kb fragment deletion within *NCgl2213*  GAS355 *ΔNCgl2277*; 0.3 kb fragment deletion within *NCgl2277*  GAS355 *ΔNCgl2358*; 0.3 kb fragment deletion within *NCgl2358*  GAS355 *ΔNCgl2382*; 0.3 kb fragment deletion within *NCgl2382*  GAS355 *ΔNCgl2449*; 0.3 kb fragment deletion within *NCgl2449*  GAS355 *Δ∆NCgl2582*; 0.3 kb fragment deletion within *NCgl2582*  GAS355 *ΔNCgl2709*; 0.3 kb fragment deletion within *NCgl2709*  GAS355 *ΔNCgl2952*; 0.3 kb fragment deletion within *NCgl2952*  pK19*mobsacB* derivative, 7.24 kb; 1.56 kb *NCgl0186* up and downstream fragments  pK19*mobsacB* derivative, 7.40 kb; 1.72 kb *NCgl0219* up and downstream fragments  pK19*mobsacB* derivative, 7.38 kb; 1.71 kb *NCgl0313* up and downstream fragments  pK19*mobsacB* derivative, 7.44 kb; 1.76 kb *NCgl0324* up and downstream fragments  pK19*mobsacB* derivative, 7.19 kb; 1.51 kb *NCgl0503* up and downstream fragments  pK19*mobsacB* derivative, 6.89 kb; 1.21 kb *NCgl0908* up and downstream fragments  pK19*mobsacB* derivative, 7.46 kb; 1.79 kb *NCgl1213* up and downstream fragments  pK19*mobsacB* derivative, 7.25 kb; 1.58 kb *NCgl1302* up and downstream fragments  pK19*mobsacB* derivative, 7.33 kb; 1.65 kb *NCgl1459* up and downstream fragments  pK19*mobsacB* derivative, 6.88 kb; 1.2 kb *NCgl1608* up and downstream fragments  pK19*mobsacB* derivative, 6.88 kb; 1.2 kb *NCgl1962* up and downstream fragments  pK19*mobsacB* derivative, 7.34 kb; 1.66 kb *NCgl2122* up and downstream fragments  pK19*mobsacB* derivative, 6.88 kb; 1.2 kb *NCgl2213* up and downstream fragments  pK19*mobsacB* derivative, 7.12 kb; 1.45 kb *NCgl2277* up and downstream fragments  pK19*mobsacB* derivative, 7.19 kb; 1.51 kb *NCgl2358* up and downstream fragments  pK19*mobsacB* derivative, 7.36 kb; 1.68 kb *NCgl2382* up and downstream fragments  pK19*mobsacB* derivative, 7.5 kb; 1.82 kb *NCgl2449* up and downstream fragments  pK19*mobsacB* derivative, 7.35 kb; 1.68 kb *NCgl2582* up and downstream fragments  pK19*mobsacB* derivative, 7.42 kb; 1.74 kb *NCgl2709* up and downstream fragments  pK19*mobsacB* derivative, 7.27 kb; 1.59 kb *NCgl2952* up and downstream fragments | This study  This study  This study  This study  This study  This study  This study  This study  This study  This study  This study  This study  This study  This study  This study  This study  This study  This study  This study  This study  This study  This study  This study  This study  This study  This study  This study  This study  This study  This study  This study  This study  This study  This study  This study  This study  This study  This study  This study  This study |

**Supplementary Table 2.** List of twenty candidate genes/proteins and primers used to screen for aromatic aldehyde reduction in this study.

| Gene identifier, protein | Primers | Sequence (5’→3’) |
| --- | --- | --- |
| NCgl 0186,  Short-chain dehydrogenase | P1  P2  P3  P4  P-C5  P-C6 | accatgattacgccaagcttgcgaaaaaccaggtgaaaaacc  caccatctgcggacatcttggccacgattgctacgtcaac  gttgacgtagcaatcgtggccaagatgtccgcagatggtg  aaacgacggccagtgaattcatagccaacaccgcaaccac  caccaaagctggccaaggat  gttcatgtccgacaagccga |
| NCgl 0219,  Zn-dependent alcohol dehydrogenase | P7  P8  P9  P10  P-C11  P-C12 | accatgattacgccaagctttgcggtgttcaaaatggggta  tgatggttcgataacccattttcattgcaacgcgatcaccaacc  ggttggtgatcgcgttgcaatgaaaatgggttatcgaaccatca  aaacgacggccagtgaattcaaaactcctggcttgagctgg  gttaaaccgcatgtgtcggg  ggaaccgcaatgatgacgtg |
| NCgl 0313,  Zn-dependent alcohol dehydrogenase | P13  P14  P15  P16  P-C17  P-C18 | accatgattacgccaagcttaaccttggtgtcttccgagtc  cgccgaattccttcgcccaggtcatcttcttagaggcgtt  aacgcctctaagaagatgacctgggcgaaggaattcggcg  aaacgacggccagtgaattcatccaggtgagtgtccaggg  gcacagcttgattccgtcac  tggtttccgctggtagatcc |
| NCgl 0324,  Zn-dependent alcohol dehydrogenase | P19  P20  P21  P22  P-C23  P-C24 | accatgattacgccaagcttcgtggtggacatcgccaagt  aacagccatgacaccgtgtgctgcgcacagcagtggtgc  gcaccactgctgtgcgcagcacacggtgtcatggctgtt  aaacgacggccagtgaattccgactgcgatgatgtaaattcc  cgacggtggcattgtctttg  tgtaaggctttccaagcggt |
| NCgl 0503,  2,5-Diketo-D-gluconic acid reductase | P25  P26  P27  P28  P-C29  P-C30 | accatgattacgccaagcttgttctccccgaaaaggtgg  gttggtggagcgtggaatcgggacttcaatcagcgtttcc  ggaaacgctgattgaagtcccgattccacgctccaccaac  aaacgacggccagtgaattcaagttcggcatgcagaagca  cggataatgccagaacaggt  aggggaaccaatcgtggc |
| NCgl 0908,  Putative multicopper oxidase | P31  P32  P33  P34  P-C35  P-C36 | accatgattacgccaagcttcaccgtgatgcccagcgc  cacggctaaagttgcggttggcctccagggcaaagtgca  tgcactttgccctggaggccaaccgcaactttagccgtg  aaacgacggccagtgaattccagcatgatgccgagctcg  gcgagcccaagaaaatagcg  ttttcgcggtgtaggaggtc |
| NCgl 1213,  Predicted oxidoreductase related to aryl-alcohol dehydrogenase | P37  P38  P39  P40  P-C41  P-C42 | accatgattacgccaagcttatcggttctgcagattctgaca  cccaacgattaatgatggaataggaaatgatcaactcatcgcggt  accgcgatgagttgatcatttcctattccatcattaatcgttggg  aaacgacggccagtgaattctcattgaagaaacagaaccaacc  ttccccggcaatgacacaat  ttgcggttcttctcgtgtgt |
| NCgl 1302,  Aldo/keto reductase related to diketogulonate reductase | P43  P44  P45  P46  P-C47  P-C48 | accatgattacgccaagctttcctcgatccagcagcgcg  tggaattgcgtgacgtctaccgccgtcgaaaagcagcttc  gaagctgcttttcgacggcggtagacgtcacgcaattcca  aaacgacggccagtgaattctcctcgacgatcccggcc  gttctgaaagccgtgacctt  gtcttttccgttttcggcgc |
| NCgl 1459,  Predicted oxidoreductase related to aryl-alcohol dehydrogenase | P49  P50  P51  P52  P-C53  P-C54 | accatgattacgccaagctttctcggccaaaatgaacacca  cgccttgccccagcggaggcagggtgacatctaattggg  cccaattagatgtcaccctgcctccgctggggcaaggcg  aaacgacggccagtgaattcccagaagctgatcatcgctg  ttggtggctacttcacgctt  atgacaggaacggcgatgag |
| NCgl 1608,  Predicted iron-dependent peroxidase | P55  P56  P57  P58  P-C59  P-C60 | accatgattacgccaagctttggaggacagcgacatgacg  ataaacaccaaaccagcattggagcgtaaacgcaaccagattc  gaatctggttgcgtttacgctccaatgctggtttggtgtttat  aaacgacggccagtgaattccttttgcacaaacgatagcctc  gatcaccacggaaaacgctg  ttttcaatggctgggccgat |
| NCgl 1962,  Glycine/D-amino acid oxidase | P61  P62  P63  P64  P-C65  P-C66 | accatgattacgccaagcttttcacggtgtgcatcttggg  gggcgggcgccgcaggagcggattccttcatcaacaaa  tttgttgatgaaggaatccgctcctgcggcgcccgccc  aaacgacggccagtgaattctcgaggcggcgcagcatg  gcatcaaaggttgcgcttga  ggcgatgacctccactttca |
| NCgl 2122,  Short-chain dehydrogenase | P67  P68  P69  P70  P-C71  P-C72 | accatgattacgccaagctttttgcttgcccagtcccagg  agggtctgctggtgggtttcttggtcagtgacatcgagtgc  gcactcgatgtcactgaccaagaaacccaccagcagaccct  aaacgacggccagtgaattccgcttatcacgaagtttcggt  cccagctgatgcctacaagt  gttactgctgcgttggcttt |
| NCgl 2213,  Hypothetical protein | P73  P74  P75  P76  P-C77  P-C78 | accatgattacgccaagcttggtccagcaccttcaaaacg  gagcgcttttcttcgtcgcttctgcaactgtggtgggctc  gagcccaccacagttgcagaagcgacgaagaaaagcgctc  aaacgacggccagtgaattcgagcgctagagccacggtg  ctgtcggcgtgccataaatc  tggaagattgcctcgctgaa |
| NCgl 2277,  Aldo/keto reductase related to diketogulonate reductase | P79  P80  P81  P82  P-C83  P-C84 | accatgattacgccaagctttaaggggttttggaatccttc  ggaagttgcacacaccgatggcgctctgcttcatcgggat  atcccgatgaagcagagcgccatcggtgtgtgcaacttcc  aaacgacggccagtgaattcgcagtaccgcgaacggttca  tgaaccatgtctcgatgcgt  gctcattttggccaccgaag |
| NCgl 2358,  Short-chain dehydrogenase | P85  P86  P87  P88  P-C89  P-C90 | accatgattacgccaagcttggacagcccgatatcagtcaa  aaggcgtcggcgagtccgtcgataggctctactccctc  gagggagtagagcctatcgacggactcgccgacgcctt  aaacgacggccagtgaattctgccctatcgccgcaattctt  cgcttccacatctgggttct  tggaacttctttccggctcc |
| NCgl 2382,  Zn-dependent alcohol dehydrogenases | P91  P92  P93  P94  P-C95  P-C96 | accatgattacgccaagctttcagatcggaccgatggcga  tgagcacgcccgcagcgcatgcgaagttgcggtcataga  tctatgaccgcaacttcgcatgcgctgcgggcgtgctca  aaacgacggccagtgaattcattcggtctgcagcggtgg  tacgcggaagtagtcgatgc  tggatagctccgaggtggaa |
| NCgl 2449,  Zn-dependent oxidoreductase | P97  P98  P99  P100  P-C101  P-C102 | accatgattacgccaagcttgcactgctccgaagttatcc  atgatgtcgcttgcgcccagcaaaccagcgccattcagca  tgctgaatggcgctggtttgctgggcgcaagcgacatcat  aaacgacggccagtgaattcaaagctagtagcgctcaggct  tgatcagctggcaaacccag  gacgccaacagtcgtgatct |
| NCgl 2582,  Diacetyl reductase | P103  P104  P105  P106  P-C107  P-C108 | accatgattacgccaagctttctacggcaagcatccggttt  gatgccaggtgcgtaggcaccaagtttctctgctgcctca  tgaggcagcagagaaacttggtgcctacgcacctggcatc  aaacgacggccagtgaattcggctattaagccagacatgtag  aaagacgaggtaagccctgc  cggcgtcataaacgagggta |
| NCgl 2709,  Zn-dependent alcohol dehydrogenase | P109  P110  P111  P112  P-C113  P-C114 | accatgattacgccaagcttgattcctattgactaggcgac  gcccatcgccgctgcgtatatatcgccgaccttcacatcg  cgatgtgaaggtcggcgatatatacgcagcggcgatgggc  aaacgacggccagtgaattcgacagctccagggattcctg  cacgttgcggacggtttaag  ctgatacagctctgaggccg |
| NCgl 2952,  Maleylacetate reductase | P115  P116  P117  P118  P-C119  P-C120 | accatgattacgccaagcttcatcatgatccttgttgttggg  tccggagcctgcggaggccagaccccacacgttggttg  caaccaacgtgtggggtctggcctccgcaggctccgga  aaacgacggccagtgaattctcaacctcaggagcgtcatc  tgccaacattgctttcggtg  attcggaacgggattgcctt |

**Supplementary Table 3.** List of primers used for gene amplification or gene deletion/insertion in this study.

| Primers | Sequence (5’→3’)^*^ | Remarks | Restriction site |
| --- | --- | --- | --- |
| P-0324-F  P-0324-R | gaagtccaggaggagaattcatgagtatctcagtaaaagcata ccgccaaaacagccaagcttctaaaccgcctcaacctcagc | Cloning of *NCgl0324*  in pCXE50 | *Eco*RI  *Hin*dIII |
| P-0324-NF  P-0324-XR | aagaaggagatatacatatgagtatctcagtaaaagcataca tggtggtggtggtgctcgagaaccgcctcaacctcagcaa | Cloning of *NCgl0324*  in pET24a | *Nde*I  *Xho*I |
| P-X48-F  P-Ptuf-mR | gagcaaaaacaggaaggcaaaat  cccggaattcaatccctcctttcctacgtggtggctacgactttcgc | Amplification of mutant  *tuf* promoter | *Eco*RI |
| P-TrrnB-tF  P-X48-R | ccccaagcttagagtagggaactgccaggca  tagaccgtatccaaagcatcc | Amplification of *rrnB*  truncated transcriptional  terminator |  |
| P-pobA-F  P-pobA-R  P-pobA-CF  P-pobA-CR | tgaccatgattacgccaagcttcaccgagcacaccaccgag  cccggggatcctctagagtactgcggggattctggaag  tcaacgaaaattgcggcacc  ctcgccgagttcttcttcgt | Amplification of *pobA*  ORF with up and  downstream regions  Confirm primers for *pobA* recovery | *Hin*dIII  *Xba*I |
| P-pca-CF  P-pca-CR | acaactgccagatcgatcacg  agcggaatccatcatcggtc | Confirm primers for  *pcaHG* partial deletion |  |
| P-vanA-F  P-vanA-R  P-vanB-F  P-vanB-R  P-van-CF  P-van-CR | accatgattacgccaagctttaccgcaccaaagatggccg  gggcccatggttcgcgctctagagatccaccttgccttcaaatcc  tctagagcgcgaaccatgggccccttctctgatcgtcaaaaagaatc  aaacgacggccagtgaattcccatgccgcggtttttgttgt  tcgccgcttgggattatgaa  ccgtaaccagcagcgtagat | Amplification of 0.556 kb *vanA* partial fragment  Amplification of 0.55 kb *vanB* partial fragment  Confirm primers for 1.3 kb *vanAB* partial deletion | *Hin*dIII  *Eco*RI |

^*^Underlines indicate restriction enzyme sites.

**Supplementary Table 4.** Nucleotide sequence of codon-optimized mutated *comt* gene from *Rattus norvegicus* encoding a mutated soluble catechol *O*-methyltransferase.

| Condon-optimized nucleotide sequence of *comt* gene |
| --- |
| **ATG**GGAGATACGAAAGAACAACGCATTCTTCGGTATGTCCAACAGAACGCCAAGCCCGGAGATCCTCAAAGCGTCTTGGAAGCAATCGACACTTACTGTACCCAGAAGGAGTGGGCTATGAATGTAGGTGACGCCAAGGGACAGATCATGGATGCCGTTATCCGGGAATACTCGCCTTCATTGGTACTGGAATTGGGCGCATACTGCGGATATAGCGCGGTACGGATGGCCCGGCTTCTCCAGCCAGGAGCGCGGTTGTTGACTATGGAAATGAATCCCGACTACGCAGCCATTACGCAGCAAATGCTGAATTTCGCCGGATTGCAGGATAAGGTGACCATCCTTAATGGCGCATCCCAGGATCTTATTCCACAGTTGAAAAAGAAGTATGACGTGGATACTCTCGATATGGTGTTTCTCGATCATTGGAAGGATCGTTATCTTCCCGATACGCTCCTCTTGGAGAAGTGTGGCCTCCTCCGCAAGGGTACTGTCCTGCTGGCGGACAACGTAATTGTTCCCGGCACTCCGGACTTCTTGGCTTATGTTCGCGGATCTTCATCTTTCGAGTGTACGCATTACTCCTCATATCTCGAGTTGATGAAAGTAGTTGACGGATTGGAAAAAGCTATTTATCAGGGTCCCTCCTCGCCTGACAAGAGC**TAA** |

**Supplementary Table 5.** List of candidate genes related to aromatic aldehyde reduction in *C. glutamicum*.

| NCgl number  Criteria | 186 | 219 | 313 | 324 | 503 | 908 | 1213 | 1302 | 1459 | 1608 | 1962 | 2122 | 2213 | 2277 | 2358 | 2382 | 2449 | 2582 | 2709 | 2952 | 99 | 168 | 689 | 992 | 1003 | 1112 | 2053 | 2902 |
| --- | --- | --- | --- | --- | --- | --- | --- | --- | --- | --- | --- | --- | --- | --- | --- | --- | --- | --- | --- | --- | --- | --- | --- | --- | --- | --- | --- | --- |
| **1. Bioinformatics analysis** | | | | | | | | | | | | | | | | | | | | | | | | | | | | |
| 1) Alcohol dehydrogenases |  | ✓ | ✓ | ✓ |  |  | ✓ |  | ✓ |  |  |  |  |  | ✓ |  | ✓ |  | ✓ | ✓ | ✓ |  |  |  |  |  |  | ✓ |
| 2) Oxidoreductases related to aryl-alcohol dehydrogenase |  |  |  |  |  |  | ✓ |  | ✓ |  |  |  |  |  |  |  |  |  |  |  | ✓ |  |  |  |  |  |  |  |
| 3) Aldo-keto reductases |  |  |  |  | ✓ |  |  | ✓ |  |  |  |  |  | ✓ |  |  |  |  |  |  |  |  |  |  | ✓ |  |  |  |
| 4) Short-chain dehydrogenases | ✓ |  |  |  |  |  |  |  |  |  |  |  |  |  | ✓ |  |  |  |  |  |  |  | ✓ | ✓ |  |  |  |  |
| 5) SDR motif [TGXXXGXG] |  |  |  |  |  |  |  |  |  |  |  | ✓ |  |  | ✓ |  | ✓ | ✓ |  |  |  |  | ✓ |  |  |  | ✓ |  |
| 6) MDR motif  [GHEX_2_GX_5_(G,A)X_2_(I,V,A,C,S)] |  | ✓ | ✓ | ✓ |  |  |  |  |  |  |  |  |  |  |  | ✓ |  |  | ✓ |  |  |  |  |  |  |  |  |  |
| **2. Literature search and homology analysis** (genes showing E-value less than E^-20^) | | | | | | | | | | | | | | | | | | | | | | | | | | | | |
| 1) Rodriguez and Atsumi (2012) | | | | | | | | | | | | | | | | | | | | | | | | | | | | |
| AdhP |  | **✓** | **✓** | **✓** |  |  |  |  |  |  |  |  |  |  |  |  |  |  | **✓** |  |  |  |  |  |  |  |  |  |
| FucO |  |  |  |  |  |  |  |  |  |  |  |  |  |  |  |  |  |  |  | **✓** |  |  |  |  |  | **✓** |  |  |
| EutG |  |  |  |  |  |  |  |  |  |  |  |  |  |  |  |  |  |  |  | **✓** |  |  |  |  |  | **✓** |  |  |
| YjgB |  | **✓** |  | **✓** |  |  |  |  |  |  |  |  |  |  |  |  |  |  | **✓** |  |  |  |  |  |  |  |  |  |
| 2) Rodriguez and Atsumi (2014) | | | | | | | | | | | | | | | | | | | | | | | | | | | | |
| YahK |  | **✓** |  | **✓** |  |  |  |  |  |  |  |  |  |  |  |  |  |  | **✓** |  |  |  |  |  |  |  |  |  |
| YbbO | (no homologous proteins) | | | | | |  |  |  |  |  |  |  |  |  |  |  |  |  |  |  |  |  |  |  |  |  |  |
| GldA | (no homologous proteins) | | | | |  |  |  |  |  |  |  |  |  |  |  |  |  |  |  |  |  |  |  |  |  |  |  |
| DkgA |  |  |  |  | **✓** |  |  | **✓** |  |  |  |  |  | **✓** |  |  |  |  |  |  |  |  |  |  | **✓** |  |  |  |
| YghA |  |  |  |  |  |  |  |  |  |  |  |  |  |  |  |  |  | **✓** |  |  |  |  |  |  |  |  | **✓** |  |
| 3) Kunjapur et al. (2014) |  |  |  |  |  |  |  |  |  |  |  |  |  |  |  |  |  |  |  |  |  |  |  |  |  |  |  |  |
| DkgA |  |  |  |  | **✓** |  |  | **✓** |  |  |  |  |  | **✓** |  |  |  |  |  |  |  |  |  |  | **✓** |  |  |  |
| YqhD | (no homologous proteins) | | | | | |  |  |  |  |  |  |  |  |  |  |  |  |  |  |  |  |  |  |  |  |  |  |
| YahK |  | **✓** |  | **✓** |  |  |  |  |  |  |  |  |  |  |  |  |  |  | **✓** |  |  |  |  |  |  |  |  |  |
| YjgB |  | **✓** |  | **✓** |  |  |  |  |  |  |  |  |  |  |  |  |  |  | **✓** |  |  |  |  |  |  |  |  |  |
| 4) Hansen et al. (2009) | | | | | | | | | | | | | | | | | | | | | | | | | | | | |
| ADH6 |  | **✓** |  | **✓** |  |  |  |  |  |  |  |  |  |  |  |  |  |  | **✓** |  |  |  |  |  |  |  |  |  |
| 4) Tsuge et al. (2016) | | | | | | | | | | | | | | | | | | | | | | | | | | | | |
| FudC |  |  |  | **✓** |  |  |  |  |  |  |  |  |  |  |  |  |  |  |  |  |  |  |  |  |  |  |  |  |
| 5) Zhou et al. (2019) | | | | | | | | | | | | | | | | | | | | | | | | | | | | |
| CGS9114_RS10340 |  |  |  |  |  |  |  |  |  |  |  |  |  |  |  |  |  |  |  |  |  | **✓** |  |  |  |  |  |  |
| CGS9114_RS09230 |  |  |  |  |  |  |  |  |  |  |  |  | **✓** |  |  |  |  |  |  |  |  |  |  |  |  |  |  |  |
| CGS9114_RS09375 |  |  |  |  |  | **✓** |  |  |  |  |  |  |  |  |  |  |  |  |  |  |  |  |  |  |  |  |  |  |
| CGS9114_RS11565 |  |  |  |  |  |  |  |  |  |  | **✓** |  |  |  |  |  |  |  |  |  |  |  |  |  |  |  |  |  |
| CGS9114_RS06005 |  |  |  |  |  |  |  |  |  | **✓** |  |  |  |  |  |  |  |  |  |  |  |  |  |  |  |  |  |  |
| CGS9114_RS01115 |  |  |  |  |  |  |  |  |  |  |  |  |  |  |  |  |  |  | **✓** |  |  |  |  |  |  |  |  |  |

**Supplementary Table 6.** Predicted translation initiation rate and total ΔG of *comt*^m^ gene in the plasmids pYL200, pYL230, and pYL250 using RBS calculator.

| Plasmid | Translation initiation rate (au) | ΔG_total_ |
| --- | --- | --- |
| pYL200 | 19388.95 | -6.12 |
| pYL230 | 3028.43 | -2.00 |
| pYL250 | 25171.93 | -6.70 |

**Supplementary Figure 1**

**
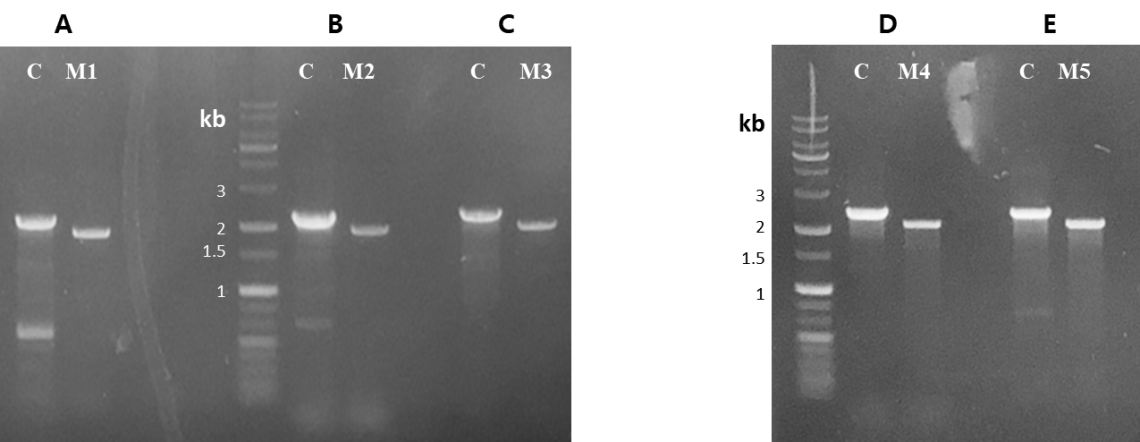
**

**
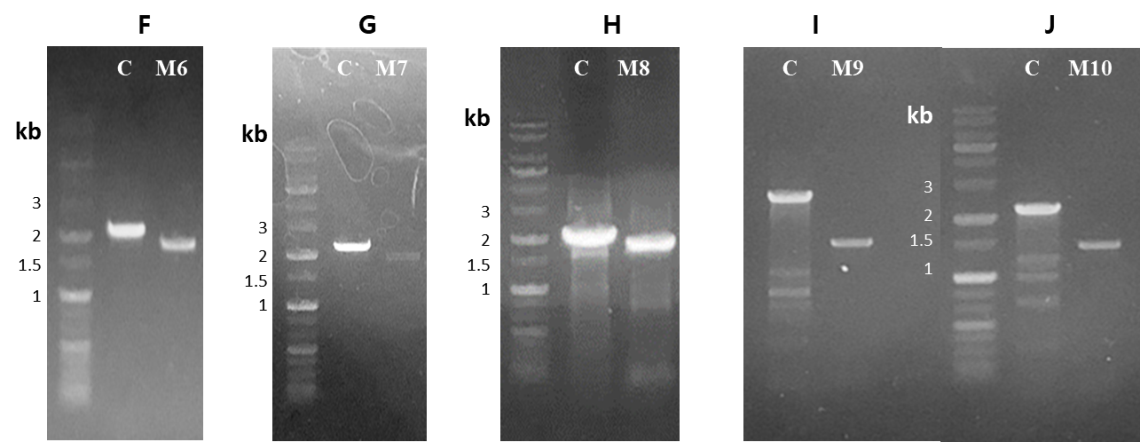
**

**
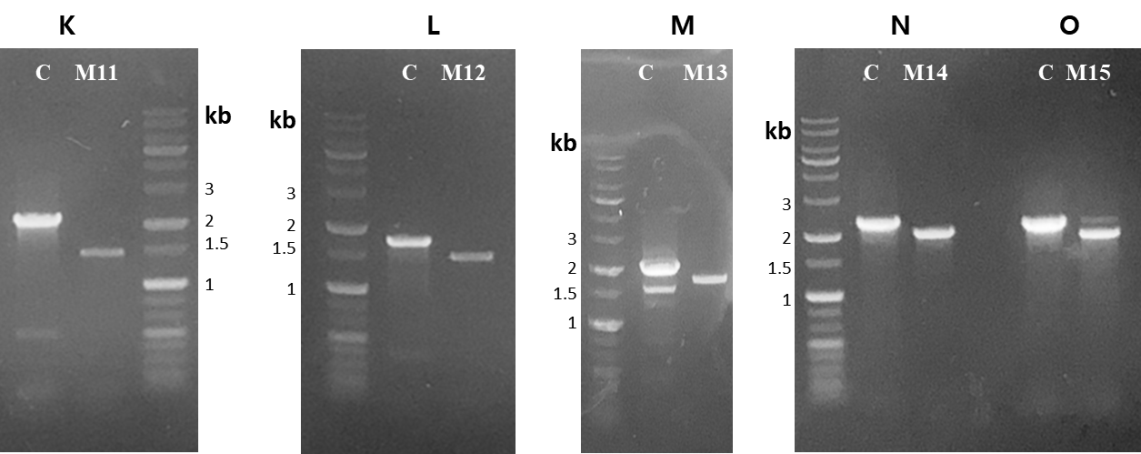
**

**
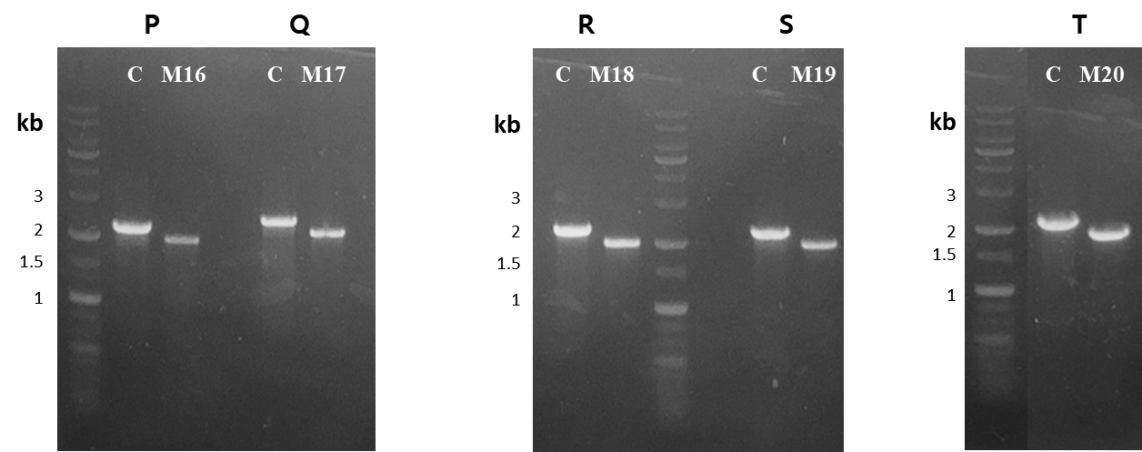
**

**Supplementary Figure 2**

**
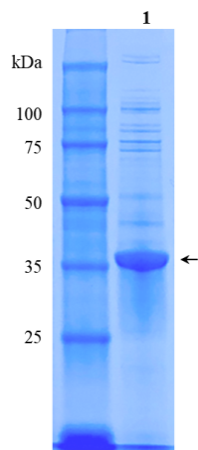
**

**Supplementary Figure 3**

**
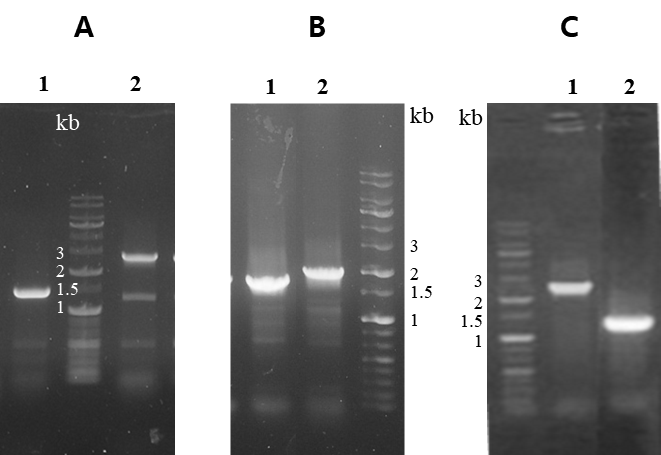
**

**Supplementary Figure 4**


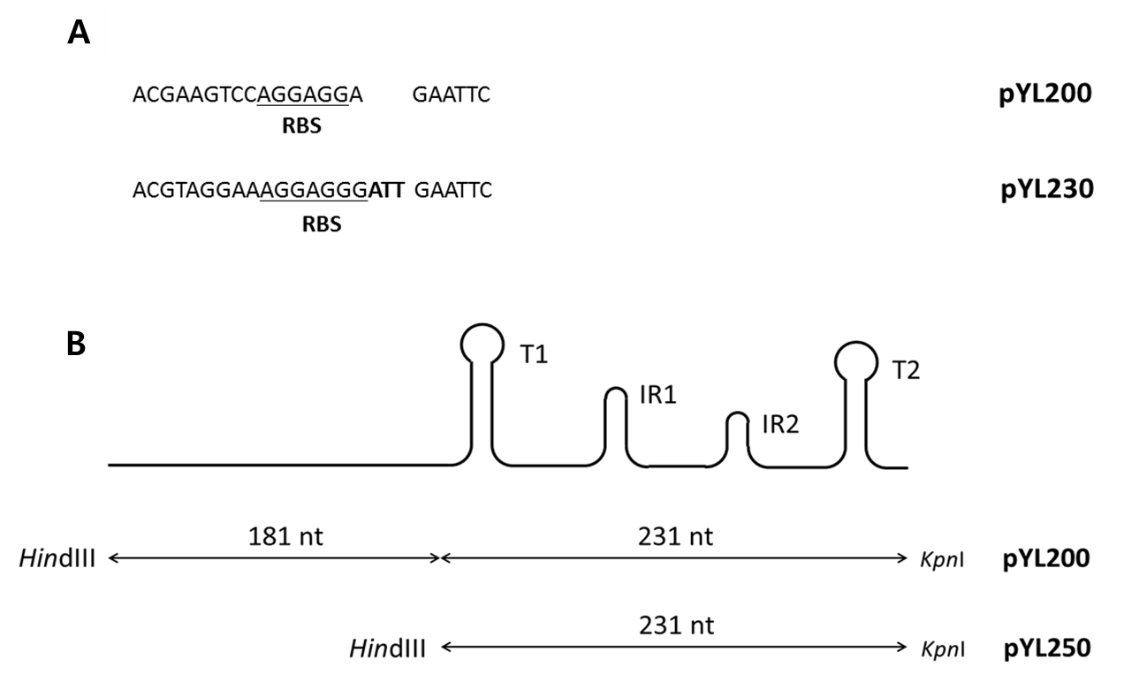


**Supplementary Figure 5**


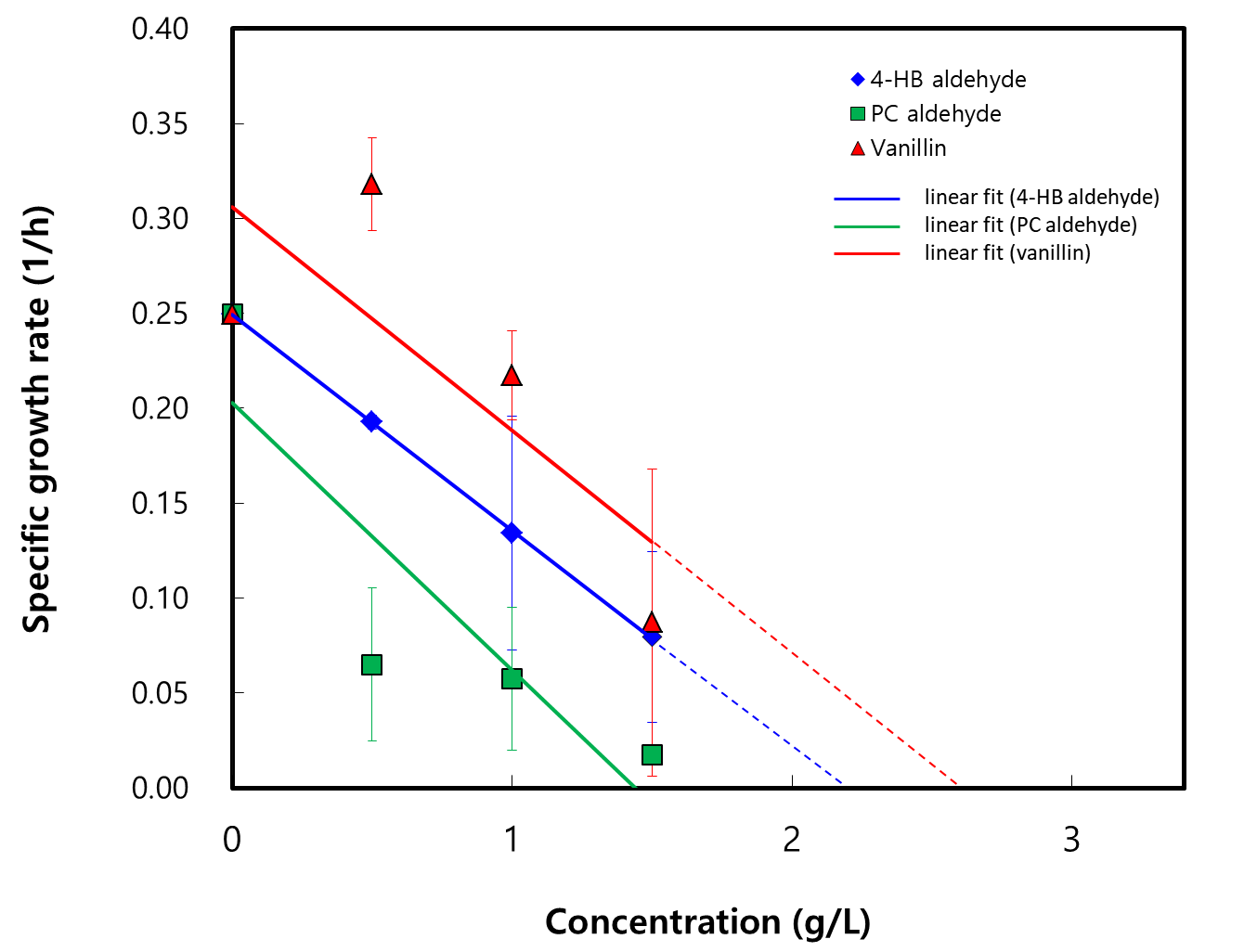

Supplement: Supplementary file 1 [file DataSheet1.docx]
